# Large Enhancement of Photoluminescence Obtained in Thin Polyfluorene Films of Optimized Microstructure

**DOI:** 10.3390/polym16162278

**Published:** 2024-08-11

**Authors:** Otto Todor-Boer, Cosmin Farcău, Ioan Botiz

**Affiliations:** 1 Research Institute for Analytical Instrumentation Subsidiary, National Institute for Research and Development of Optoelectronics Bucharest INOE 2000, 67 Donath Street, 400293 Cluj-Napoca, Romania; otto.todor@icia.ro; 2 National Institute for Research and Development of Isotopic and Molecular Technologies INCDTIM, 67-103 Donath Street, 400293 Cluj-Napoca, Romania; cosmin.farcau@itim-cj.ro; 3 Interdisciplinary Research Institute on Bio-Nano-Sciences, Babeș-Bolyai University, 400271 Cluj-Napoca, Romania; 4 Department of Physics of Condensed Matter and Advanced Technologies, Faculty of Physics, Babeș-Bolyai University, 400084 Cluj-Napoca, Romania

**Keywords:** polyfluorenes, solvent vapor annealing, film microstructure, photoluminescence, atomic force microscopy

## Abstract

There is a clearly demonstrated relationship between the microstructure, processing and resulting optoelectronic properties of conjugated polymers. Here, we exploited this relationship by exposing polyfluorene thin films to various solvent vapors via confined-solvent vapor annealing to optimize their microstructure, with the final goal being to enhance their emission properties. Our results have demonstrated enlargements in photoluminescence intensity of up to 270%, 258% and 240% when thin films of polyfluorenes of average molecular weights of 105,491 g/mol, 63,114 g/mol and 14,000 g/mol, respectively, experienced increases in their *β*-phase fractions upon processing.

## 1. Introduction

Conjugated polymers have attracted considerable attention in recent years for various reasons. These materials are less and less expensive, have a high flexibility and low weight, exhibit good solubility and processability, and thus, are highly suitable for a plethora of organic devices and applications [1,2,3,4,5,6]. Moreover, such applications are feasible because conjugated polymers exhibit a large variety of optoelectronic properties, such as charge separation, transport and recombination, and the absorption and emission of light, just to name a few [7,8,9,10,11,12,13,14]. These properties are greatly influenced by the now well-known microstructure–processing–optoelectronic properties relationship that dictates the final molecular arrangements of polymeric chains at the nano- and microscale, and correlates them with the resulting properties of the conjugated material [15,16,17,18,19,20,21].

Polyfluorenes such as poly (9,9-di-n-octylfluorenyl-2,7-diyl) (PFO), are conjugated polymers composed of repeating units of fluorene linked together through polymerization [22,23], and are no exception as regards the above relationship. Therefore, PFOs’ optoelectronic properties [24,25,26], widely exploited in domains such as energy conversion [27], lighting [28,29] (PFO-based light-emitting diodes can emit at a variety of wavelengths, including white [30], red [31], green [32] or blue light [33,34]), field-effect transistors [35], organic solar cells [36,37], or displays and sensors [38,39,40,41,42,43,44,45], will strongly depend not only on the chemical structure of the constituent monomers, but also on the molecular arrangements adopted by the PFO chains within the film microstructure. Thus, all the above-mentioned PFO-based applications, and especially the organic light-emitting diodes, are limited not only to the continuous design and development of new PFO derivatives, but also to the advancement of material processing methods able to establish control over the molecular arrangements both at the nano- and microscale.

The two most widely encountered molecular arrangements exhibited by PFO systems include amorphous/glassy and *β*-phase conformations [46,47,48,49,50,51]. While in the glassy phase the PFO chains tend to arrange within disordered wormlike conformations exhibiting a broad distribution of intermonomer torsion angles [51,52,53], the *β*-phase is usually comprised of PFO molecules displaying an extended, rather coplanar geometry characterized by a torsion angle between the adjacent fluorene units ranging between 165° and 180° [51,52,53,54,55,56,57]. The exact fraction of these phases in a specific PFO solution or film is generally highly dependent on the chemical structure of monomers, molecular weight, type of utilized solvent or processing methods [58,59,60,61]. For instance, thin PFO films composed of separated glassy and *β*-phase regions, with the *β*-phase being rather concentrated in the aggregates, can nowadays be routinely obtained [62,63,64].

There is a large variety of processing methods that are specific to solutions, films or bulk materials [65], each exhibiting advantages and disadvantages. Nonetheless, one of the simplest ways to alter the film microstructure, and thus the optoelectronic properties of thin films based on conjugated polymers such as PFO, is to expose these films to solvent vapors after their fabrication [66,67,68,69] (it was demonstrated that the *β*-phase can be obtained after thin film fabrication by exposing the glassy phase films to solvent vapor [62] or thermal [70] annealing). This way, the mobility of PFO chains can be increased, and the latter can start ordering and/or adopting more extended/planarized chain conformations [71,72,73]. Nonetheless, to be highly efficient, this technique might require a more precise mode of control over the evaporation rate and a rich swelling of the films [74].

Here, we use a film post-deposition processing technique called space-confined solvent vapor annealing (C-SVA) to alter the microstructures of poly(9,9-di-n-octylfluorenyl-2,7-diyl) (of average molecular weights of M_w_ = 105,491 g/mol, M_w_ = 63,114 g/mol and M_w_ = 14,000 g/mol; hereafter simply PFO_105k_, PFO_63k_ and PFO_14k_, respectively) systems, and thereby to enhance their emission properties in the thin film configuration. The C-SVA technique has been successfully applied before to achieve the self-assembly [74] and crystallization [75] of diverse copolymers [76] and biopolymers [77], and it is based on the abundant precipitation of solvent vapors onto the thin polymer films. The precipitation usually induces a rich swelling of films and provides polymer chains with high mobility. The subsequent slow and highly controlled evaporation of solvent vapors further favors polymer chains to order and/or to adopt more highly extended or planarized chain conformations.

As the goal of this study was to employ an efficient processing method to alter the primal molecular arrangements adopted by various PFO chains upon their spin casting into thin films, and thus to significantly enhance their resulting emission properties, we have employed the C-SVA approach and the idea of rich swelling of thin polymeric films through their exposure to solvent vapors [78,79]. As a consequence, the results obtained by UV-Vis absorption, atomic force microscopy (AFM) and Raman spectroscopy have demonstrated that PFO films processed via the C-SVA method undergo alterations in their molecular arrangements, with more and more chains adopting the rather extended and planarized *β*-phase conformations. As revealed by the emission measurements, these structural changes massively increased the overall photoluminescence (PL) of all C-SVA-processed PFO films. Such results could be utilized, for example, when designing and developing novel, possibly more efficient organic light-emitting devices.

## 2. Materials and Methods

PFO of an average weight molecular weight M_w_ = 14,000 g/mol (hereafter simply PFO_14k_) was purchased from Sigma-Aldrich Ltd. PFOs of molecular weights of M_w_ = 63,114 g/mol (polydispersity index PDI = 3.3; PFO_63k_) and M_w_ = 105,491 g/mol (polydispersity index PDI = 2.33; PFO_105k_) were purchased from Ossila Ltd. (Sheffiled, UK). Solutions of the above polymer systems were prepared by dissolving 6 mg of PFO powder in 1 mL of toluene solvent, followed by gentle stirring at 70 °C for 10–30 min. We have chosen the type of solvent as a function of its abilities to completely dissolve the initial PFO powder and also to display an increased volatility necessary for the C-SVA processing experiments.

Thin films, of an average thickness of about 74 ± 5 nm (measured by the AFM technique, after gently scratching the films), were obtained by spin-casting the PFO solutions onto UV-ozone cleaned silicon wafers (type 4P0/5–10/380 ± 15/SSP/TTV *<* 5 from Siegert Wafer) and microscope glasses, using a spin coater (from Laurell Technologies Corporation, North Wales, PA, USA) running at a speed of 2000 rpm for 30 s. Each resulting PFO thin film was cut into two pieces. For each case, one half was always used as a reference, and the other half was further processed using the C-SVA tool. While the glass substrate configuration was needed for the absorption and PL measurements, the silicon wafers were necessary for the visualization of the interference colors within the C-SVA setup during the processing of PFO films (see more details on the interference phenomenon and procedures to determine the concentration of polymer in rich-swollen films elsewhere [78]).

The C-SVA tool was composed of an aluminum sample chamber with a Peltier element (15.4 V/8.5 A from Stonecold) at the bottom, which controlled the temperature of the bottom of the chamber to an accuracy of 0.01 °C using a special temperature controller (TCM U 10 A from Electron Dynamics Ltd.; Southampton, UK), a PT100 sensor and corresponding software. To introduce (organic) solvent vapor into the sample chamber, a gas bubbler was used. The bottom of the chamber, on which the PFO film was placed, was then cooled at a rate of 0.3 °C/s until the solvent vapors started to precipitate on the surface of the film. Consequently, the thin film became enriched and swollen and PFO chains were fully dissolved and free to move in the newly created “film solution”. The latter was then slowly heated (at a rate of only 0.01 °C/s) and the solvent gradually evaporated, causing the PFO molecules to adopt rather planarized and more extended molecular arrangements specific to *β*-phase conformations. The whole process could be monitored through a glass window on the cover of the aluminum sample chamber via an optical microscope or by the naked eye. A heat sink and a fan assisted in smoothly reaching and maintaining an accurate and yet stable sample temperature over time (between a few seconds and several hours). More detailed information on this experimental setup, as well as on the film processing procedure, can be found elsewhere [74,75,76,78].

PL spectra were collected using an FP-6500 Spectrofluorometer from Jasco (excitation wavelength range of 220–750 nm). All PL spectra were recorded using an excitation wavelength of 390 nm (each sample was measured in three different positions to make sure our data were reproducible within the 1–2% measurement error attributed to the spectrofluorometer). This excitation wavelength was inferred from the corresponding absorption spectra acquired in advance using a V-530 UV-VIS spectrophotometer from Jasco (spectral range of 190–1100 nm). To simply observe the thin films of PFOs under UV light, a UV lamp from Biocomp composed of two LBA15WT8 Teflon tubes was utilized. Raman measurements were performed using a confocal Raman Microscope Alpha 300 from Witec, equipped with a 100 × (0.9 NA) objective, matched to a 100 µm optical fiber pinhole. A power of 8 mW at the sample, provided by a 532 nm laser, was used for excitation, while the integration time was 60 s.

For the acquisition of the AFM images, a system from Molecular Devices and Tools for Nano Technology mounted on an Olympus IX71 optical microscope in tapping mode was used. The whole AFM system was acquired from Spectrum Instruments Ltd. (Limerick, Ireland). The AFM measurements were conducted by utilizing high-resolution Noncontact Golden Silicon probes from NT-MDT. Such probes had a tip height of 15 ± 1 μm and were coated with gold on the detector side cantilever. The cantilever had a length of 120–130 μm and displayed a resonance frequency in the range of 187–230 kHz. Its nominal force constant was in the range of 1.45–15.1 Nm^−1^. AFM images (256 × 256 lines) were recorded using a scanning speed of about 2 μm/s and a set point ranging between 9 and 12 V. The latter was continuously adjusted in order to maintain a very soft tapping regime.

## 3. Results and Discussion

**Figure 1:**
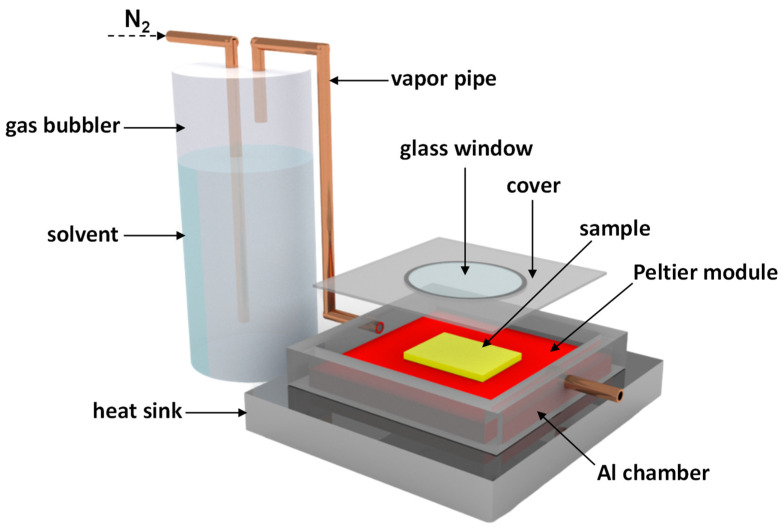
Schematic representation of the C-SVA home-made device. This device is mainly composed of a sample chamber made of aluminum and equipped with a PT100 temperature sensor. Beneath there are placed a Peltier element and a heat sink. This ensemble is connected to a temperature controller, a power source and a gas bubbling system. The latter is able to introduce a regulated amount of solvent vapors into the sample chamber.

**Figure 2:**
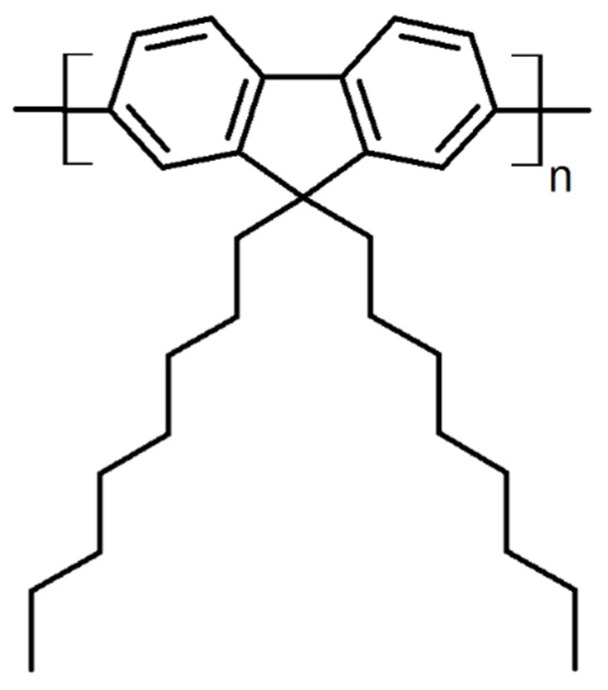
Chemical structure of PFO.

C-SVA experiments were conducted using a home-made device (schematically depicted in Figure 1) on thin films of PFO exhibiting three different molecular weights (see the chemical structure of PFO system in Figure 2). More precisely, each spin-cast PFO film was cut in two pieces, and while one half was kept as a reference sample, the other half was processed via the C-SVA method to alter its microstructure. Then, PL measurements were conducted on both samples and further compared.

**Figure 3:**
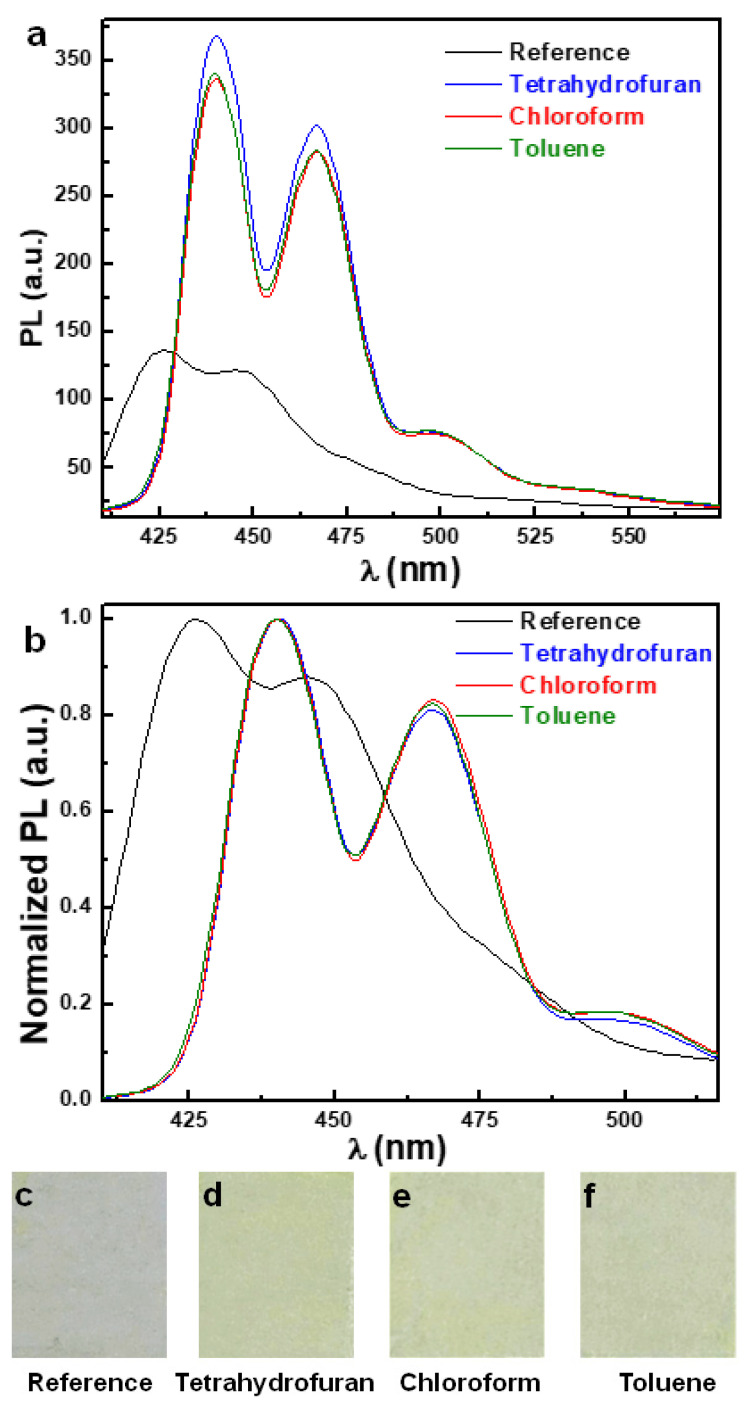
PL (**a**) and normalized PL (**b**) spectra of PFO_105k_ thin films before (black) and after their processing via the C-SVA method in tetrahydrofuran (blue), chloroform (red) and toluene (olive) vapors, along with their corresponding digital images (**c**–**f**), respectively. All PL spectra were acquired using an excitation wavelength of 390 nm.

The PL spectra obtained for thin films made of the longest PFO_105k_ chains before and after their exposure via the C-SVA method to three different solvent vapors are presented in Figure 3. One can clearly observe that all C-SVA-processed PFO_105k_ films displayed a PL intensity that was massively increased in comparison to that of the unprocessed reference film. For instance, the thin PFO_105k_ film that was exposed to tetrahydrofuran vapors exhibited an increase in PL intensity of over 270%. This value was deduced for the shorter wavelength (SW) vibronic λ_0-0_ peak located at around 426 nm (but red-shifted after the processing of the film) by dividing the maximum PL intensity of SW peaks corresponding to the C-SVA-processed and reference PFO_105k_ samples, respectively. At the same time, the enlargement of the whole area under the PL curve recorded for the C-SVA-processed PFO_105k_ film increased by about 182% when compared to the reference unprocessed film (Figure 3a). A similar behavior was also noticed for PFO_105k_ films exposed to toluene and chloroform solvents. In these cases, the PL intensities corresponding to the SW λ_0-0_ peak increased by over 250% and 247%, respectively, while the increase in the area under the PL curves was of about 174% and 170% (Figure 3a).

This massive increase in the emission intensity could further be observed by the naked eyes, as is shown in the digital images presented in Figure 3c–f. Here, we can see how an unprocessed greyish film changed its color to a brighter yellow-greenish appearance after undergoing C-SVA processing. To demonstrate that these digital images were taken in the same conditions, we have attached, in Figure S1 in the Supporting Information (SI), the original digital photograph picturing all four films in fair comparison conditions. Additionally, we have added digital photographs of two unprocessed (i.e., reference) and C-SVA-processed PFO_105k_ films that were both placed under a UV lamp (Figure S1e–f), to further emphasize that C-SVA-processed film is more emissive. Note that while we have illuminated the PFO_105k_ films with UV light of a wavelength of 253.7 nm, these films were shown to mainly absorb light at 397 nm (see Figure S2 in the SI). Therefore, while the emissive difference between the C-SVA-processed and reference PFO_105k_ films was clearly observable, this difference, as expected, was not that dramatic. Moreover, after the normalization of all PL spectra, one could better observe that the SW vibronic λ_0-0_ peak has red-shifted from 426 nm to around 440 nm once the PFO_105k_ films were exposed via the C-SVA method to three different solvent vapors (Figure 3b). A similar red-shift from 445 nm to 467 nm was also detected for the longer-wavelength (LW) λ_0-1_ vibronic peak. Finally, all PFO_105k_ films exposed to the three types of solvent vapors displayed a third emission peak at an even longer wavelength, located at around 500 nm (more visible in Figure 3b). Moreover, both PL emission peaks at SW and LW exhibit a narrower appearance and were better separated after the C-SVA processing, indicating more order in the PFO_105k_ films that were exposed to solvent vapors, as compared to the unexposed reference film. By determining that the amounts of PFO chains planarized in *β*-phase conformation dictates the PL efficiency [49,50], and taking into account that a spectral red-shift is generally indicative of structures composed of chains adopting more planar and torsionally less distorted conformations [80], we have assigned this massive enhancement of PL, observed upon the exposure of thin PFO_105k_ films to different solvent vapors, to the increase in the *β*-phase fraction upon C-SVA processing. This claim is further supported by the fact that the PL spectra shown in Figure 3 were dominated by the vibronic peaks located at 440 nm, 467 nm and 500 nm (this latter greenish emission peak, although initially assigned to interchain emission, seems to be inclusively a result of fluorenone defects [81,82]), which according to the literature are indicative of *β*-phase conformations [33,51,83,84] formed during the slow evaporation of solvent [81], with no contributions attributed to the glassy/amorphous PFO chain conformations [84]. The above claim is also sustained by the UV-vis measurements presented in Figure S2 in the SI. Here, we can clearly see that the exposure of a PFO_105k_ thin film to toluene vapors induced, besides the dominant absorption peak at 397 nm corresponding to the disordered glassy phase [33,51,83], a second absorption peak located at a longer wavelength of 431 nm and known to coincide with the *β*-phase conformations [33,50,51,81,83,85,86,87]. This second peak was rather small because the contribution of *β*-phase chains to the total absorption is known to be typically very weak [50,85].

Furthermore, the main absorption peak appeared red-shifted by ~6 nm with respect to the peak position determined for the reference film, further pointing towards an increase in the chain conjugation length induced upon C-SVA processing (note that no significant changes in the absorption intensity were noticed before and after the processing of thin films) [80]. Therefore, we conclude that by exposing thin films of PFO_105k_ to various solvent vapors via C-SVA processing, we increased the fraction of more planarized and extended *β*-phase conformations, which in turn induced a massive increase in *β*-phase- (and not glassy [83]) dominated PL at visibly higher intensities and longer wavelengths.

**Figure 4:**
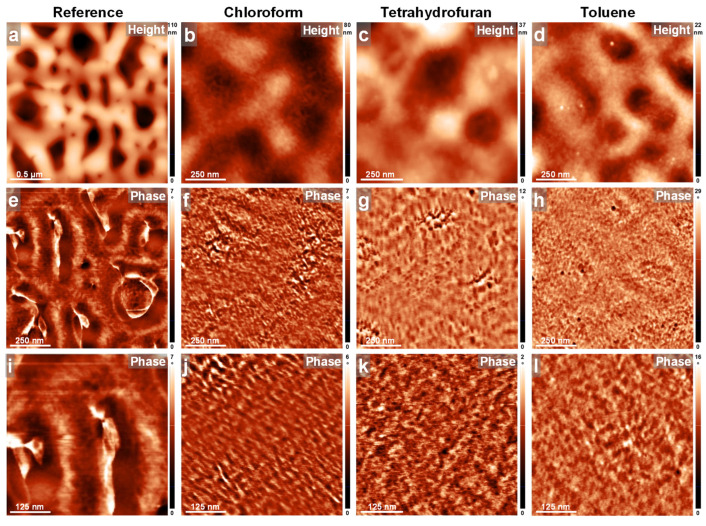
(**a**–**l**) AFM height (**a**–**d**) and phase (**e**–**l**) micrographs depicting the morphology of PFO_105k_ thin films before (**a**,**e**,**i**) and after their exposure via the C-SVA method to chloroform (**b**,**f**,**j**), tetrahydrofuran (**c**,**g**,**k**) and toluene (**d**,**h**,**l**) vapors.

To further demonstrate this narrative, we conducted AFM measurements on all PFO_105k_ films before and after their exposure via the C-SVA method to different solvent vapors. The obtained results are summarized in Figure 4. Here, we start our discussion with the reference spin cast sample, which exhibited a microstructure with a porous-like appearance, with pores measuring a few hundreds of nanometers in the lateral dimension and surrounded by higher areas (Figure 4a). As demonstrated by the AFM phase contrast (Figure 4e), these areas were highly amorphous and “felt” rather sticky during the AFM measurements. Moreover, there were almost no signs of structures of molecular dimensions when zooming-in with the AFM (Figure 4i). These results clearly emphasize the idea of a dominant amorphous morphology characterizing the surface of the spin-cast PFO_105k_ film, and thus, further support the results revealed by the absorption spectrum dominated by the large glassy peak at 397 nm [33,51,83] (see Figure S2 in the SI). Instead, PFO_105k_ films that were exposed to chloroform, tetrahydrofuran and toluene vapors via the C-SVA method also exhibited a porous-like surface (Figure 4b–d; also see Figure S3b–d,f–h in the SI). The average depths of the pores found in the reference unexposed PFO_105k_ film and in PFO_105k_ films exposed to chloroform, tetrahydrofuran and toluene vapors were measured to be around 85 ± 18 nm, 66 ± 15 nm, 64 ± 15 nm and 69 ± 16 nm, respectively (the decrease in the number and depth of such pores after the C-SVA processing could be explained by the fact that in this latter case, the evaporation of solvent vapors was well controlled, as compared to the rapid and uncontrolled evaporation of solvent vapors upon a typical spin-casting process). As further revealed by the AFM phase contrast images (see Figure 4f–h,j–l in the SI), the pores were preponderantly filled with and surrounded by more rigid/crystalline structures. Furthermore, when zooming-in with the AFM (Figure 4j–l), the surface of the PFO_105k_ films appeared to be covered with substructures of molecular dimensions such as parallel “lamellar” objects of an average width of 15 ± 3 nm (for PFO_105k_ films exposed to chloroform vapors), or more irregular features of around the same lateral dimension (for PFO_105k_ films exposed to tetrahydrofuran and toluene vapors, respectively). These smoother, yet more crystalline, morphologies seemed typical for the *β*-phase conformations [51,88,89], and fully support the appearance of the much smaller absorption peak at 431 nm (Figure S2 in the SI) representing a more crystalline phase [33,51,83,85,86,87].

**Figure 5:**
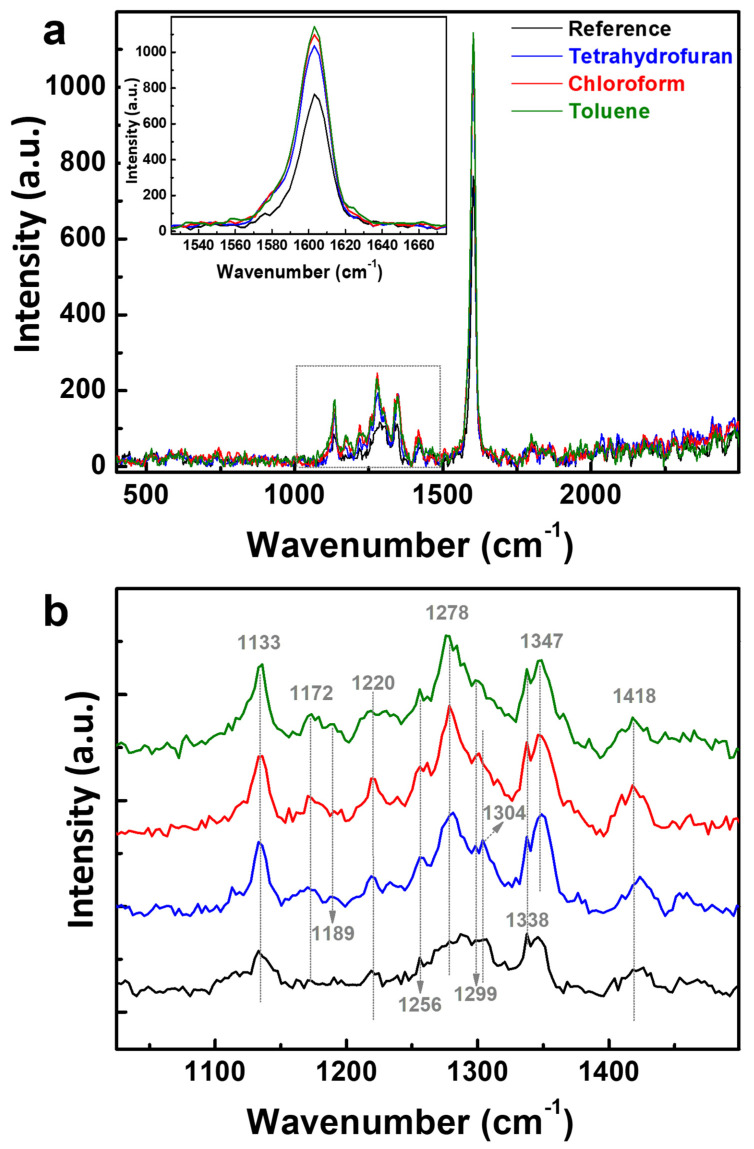
(**a**) Raman spectra recorded for a spin-cast film of PFO_105k_ before (black) and after its processing via the C-SVA method in tetrahydrofuran (blue), chloroform (red) and toluene (olive) vapors, respectively. The inset depicts a zoom-in of the main Raman peak located at around 1604 cm^−1^. (**b**) The same vertically translated Raman spectra zoomed-in in the 1000–1500 cm^−1^ spectral interval, as indicated by the dotted shape in (**a**), emphasizing the changes of various peaks induced upon C-SVA processing. Grey vertical dotted lines/arrows in (**b**) are for guiding the eye only.

In order to further strengthen our evidence on the alteration of the glassy phase and its partial transformation upon C-SVA processing into an (additional) *β*-phase, we performed Raman measurements on all PFO_105k_ thin films before and after their exposure to three types of solvent vapors. The obtained results are shown in Figure 5. When performed on PFO systems, Raman spectroscopy usually involves two types of main vibration bands. The first of them is located in the low-wavenumber region ranging between 100 cm^−1^ and 1000 cm^−1^ and corresponds to the PFO alkyl side-chain motion [90]. As this Raman spectral region did not exhibit visible differences between the unprocessed and C-SVA-processed PFO_105k_ films, we will not discuss it further here. Instead, the second band that ranges within the higher wavenumber region between 1000 cm^−1^ and 1650 cm^−1^ and represents the fluorene unit motion in the main chain (i.e., the vibrations of fluorene backbone) [90] displayed various interesting vibrational differences related to the *β*-phase conformations. The most visible peak within this spectral region was the main vibronic peak located at around 1604 cm^−1^, with a small shoulder at 1580 cm^−1^ (see the inset in Figure 5a). Generally, the Raman peak at 1600–1606 cm^−1^ is attributed to the symmetric phenyl intra-ring C–C stretching mode [91,92], and thus, an intensification of this mode is indicative of a strong increase in *β*-phase fraction [52]. Another important observation when analyzing the Raman bands was related to the intensity of the peaks in the spectral range of 1100 cm^−1^ to 1400 cm^−1^, which increased upon the C-SVA-processing of all PFO_105k_ films (see the region delimited by the dotted box in Figure 5a; see also Figure 5b and Figure S4 in the SI). As shown by previous studies, these Raman bands in the 1100–1400 cm^−1^ spectral region (including also the peaks located at 1117 cm^−1^, 1133 cm^−1^, 1172 cm^−1^, 1189 cm^−1^ and 1220 cm^−1^) could be attributed to the C–C stretching modes between the phenylene rings and C–H in-plane bending modes [90], and the increase in peak intensity means that the fraction of *β*-phase in PFO has increased [52]. As a result, the main PFO_105k_ chains became more planar. This, most probably, favored the delocalization of π electrons along the conjugated backbone and the electronic polarizability of the chain segment [93,94].

Furthermore, two distinct bands at 1256 cm^−1^ and 1278 cm^−1^ clearly developed upon the C-SVA processing of PFO_105k_ thin films in all three types of solvent vapors (Figure 5b). While these bands were previously assigned to a combination of in-plane C–H bending and to C–C stretching motions of the bond connecting the two phenylene rings within the fluorene moiety [92,95], the intensity of the peak at 1256 cm^−1^ is known to be highly sensitive to the planarity of the PFO main chain (i.e., to the intermonomer torsion angle) [52]. Thus, the development of the latter further demonstrated that the chain conformation has transformed from a less planar into a more planar one. Also, we add here the possibility of further signaling the C–C stretching modes between phenylene rings using the 1298–1314 cm^−1^ vibration bands [92,95] (usually, C–C stretching between phenylene rings belonging to the same monomeric unit is expected to occur at higher energies than that between the phenylene rings belonging to two adjacent monomeric units [92]). Finally, the 1335–1365 cm^−1^ vibronic band that intensified upon C-SVA processing could be assigned to the C–C intra-ring symmetric stretching vibrations [95]. Overall, all the above Raman results indicate that the additional *β*-phase conformations were indeed induced upon the C-SVA processing, and were clearly more planar than the primal glassy phase conformations [90], in agreement with the absorption, PL and AFM measurements presented previously.

In order to observe whether there is an effect of the length of PFO chains on resulting emission properties upon processing, we have performed similar experiments to those described above on two PFO systems of smaller molecular weights: PFO_63k_ and PFO_14k_. For an easier comparison of all PFO systems, we have extracted the PL data from Figure 3, Figures S5 and S6, and summarized them in Table 1. Alongside, we have also inferred absorption data from Figures S2, S7 and S8. Therefore, Table 1 contains not only the wavelengths of the absorption peaks corresponding to the glassy (λ_g_) and *β*-phase (λ_β_) molecular arrangements before and after their processing via the C-SVA method in the three types of solvent vapors, but also the wavelengths of their corresponding PL vibronic peaks (including λ_0-0_ and λ_0-1_), along with the PL increase ratios corresponding to the total area under the PL curves, to λ_0-0_, and to λ_0-1_ emission peaks. A careful analysis of the data suggests that tetrahydrofuran and toluene are the most suitable solvents when targeting a large enhancement in PL in thin films made of longer PFO_105k_ and PFO_63k_ chains via C-SVA processing, while chloroform seems to be more suitable for the processing of the shortest PFO_14k_ system. Moreover, the highest red-shift in the absorption spectra (~6 nm) was induced in thin films of longer PFO_105k_. This was further reflected in the higher PL enhancements induced in PFO_105k_ chains showing a longer conjugation length and adopting more planarized *β*-phase conformations. To support these conclusions, a more detailed analysis follows below.

Figures S5 and S6 show the PL spectra recorded for thin PFO_63k_ and PFO_14k_ films, before and after their exposure via the C-SVA method to various solvent vapors. As in the case of PFO_105k_ films, all C-SVA-processed PFO_63k_ and PFO_14k_ films exhibited a significantly increased PL intensity with respect to that of the unprocessed reference sample. For instance, we have deduced from Figure S5a that, while the thin PFO_63k_ film that was exposed to tetrahydrofuran vapors showed an increase in PL intensity corresponding to the SW vibronic λ_0-0_ peak of over 258% (meanwhile, the increase in the area under the PL curve was about 165%), the increase in PL corresponding to the film processed in toluene was slightly lower (253%). An even lower enhancement in PL (240%) was observed for the PFO_63k_ film exposed to chloroform (note that the increases in the area under the PL curves were about 167% and 161% for films processed in toluene and chloroform, respectively). Instead, PFO_14k_ films that were exposed to toluene, chloroform and tetrahydrofuran vapors showed increases in PL intensity corresponding to the SW vibronic λ_0-0_ peak of over 240%, 229% and 206%, respectively (meanwhile, the increases in the area under the corresponding PL curves were about 235%, 266% and 250%, respectively) (Figure S6a).

Performing the normalization of all PL spectra of PFO_63k_ films revealed that the SW vibronic λ_0-0_ peak at 424 nm has red-shifted to around 439–440 nm after C-SVA processing in all three solvents (Figure S5b). A similar red-shift from 445 nm to 466–467 nm was also observed for the LW λ_0-1_ vibronic peak (note that all C-SVA-processed PFO_63k_ films exhibited a third emission peak at 500 nm; Figure 3b). In this case, too, both PL emission peaks at SW and LW exhibited a narrower appearance and were better separated after the C-SVA processing, indicating that there was more order in the PFO_63k_ films that were exposed to solvent vapors, as compared to the unexposed reference film. As previously discussed, we have assigned this increase in PL intensity to the increase in the *β*-phase fraction upon the C-SVA processing of PFO_63k_ films. Remember that the PL spectra post-C-SVA processing were overlapping with those corresponding to *β*-phase conformations [33,51,83,84], with no contributions attributed to the glassy PFO phase [84]. Also, note that additional UV-Vis measurements (presented in Figure S7 in the SI) have indicated that the exposure of a PFO_63k_ film to toluene vapors led to a spectrum composed of a dominant absorption peak at 397 nm generated by the glassy phase [33,51,83], and a second small absorption peak located at 431 nm and representing the *β*-phase conformations [33,50,51,81,83,85,86,87]. Moreover, the appearance of a 5 nm red-shift of the main absorption peak (from 392 nm to 397 nm) upon C-SVA processing suggested that PFO_63k_ chains experienced an increase in the conjugation length by becoming more planarized and by adopting more extended *β*-phase conformations. The latter were responsible for the observed increase in PL at longer wavelengths.

As expected, the normalized PL spectra of PFO_14k_ films emphasize that the SW vibronic λ_0-0_ peak at 424 nm red-shifted to around 439–440 nm after the C-SVA processing in all three solvents (Figure S6b), with a similar red-shift from 444 nm to 466–467 nm being also observed for the LW λ_0-1_ vibronic peak (additionally, the C-SVA-processed PFO_14k_ films also exhibited a third emission peak at 500 nm). Again, the increase in PL was due to the increase in the *β*-phase fraction upon C-SVA processing, as demonstrated inclusively by the existence of an absorption peak at 399 nm and a peak shoulder at 430 nm, corresponding to the glassy and *β*-phases, respectively (see Figure S8 in the SI). Again, the red-shifting of the main absorption peak from 393 nm to 399 nm upon the C-SVA processing in toluene vapors points towards PFO_14k_ chains adopting more planarized and extended *β*-phase conformations with increased conjugation length, i.e., towards chains able to emit light more efficiently. Nonetheless, the fraction of such chains was probably lower than those corresponding to the previous cases of PFO_105k_ and PFO_63k_ chains, as indicated by the weaker absorption peak (rather, a shoulder) at 430 nm (compare Figure S8 with Figures S7 and S2 in the SI).

Once again, the increase in the emission intensity of thin PFO_63k_ and PFO_14k_ films was visible by the naked eye, as is shown in the digital images taken in the same conditions for thin PFO_63k_ films (see their fair comparison in Figure S9 in the SI) and PFO_14k_ films (see Figure S10 in the SI), respectively. For instance, in the former images, we could observe a change in the film color from grey to yellowish when spin-cast films underwent further C-SVA processing in various solvent vapors (Figures S5c–f and S9a–d). Moreover, digital photographs of two unprocessed (i.e., reference) and C-SVA-processed PFO_63k_ films that were both placed under a UV lamp (Figure S9e–f) further demonstrate that the C-SVA-processed film was clearly more emissive, although the PFO_63k_ films were both illuminated with UV light of a wavelength of 253.7 nm, and they did not absorb that much light (their main absorption peak is located at 397 nm; see Figure S7 in the SI).

**Figure 6:**
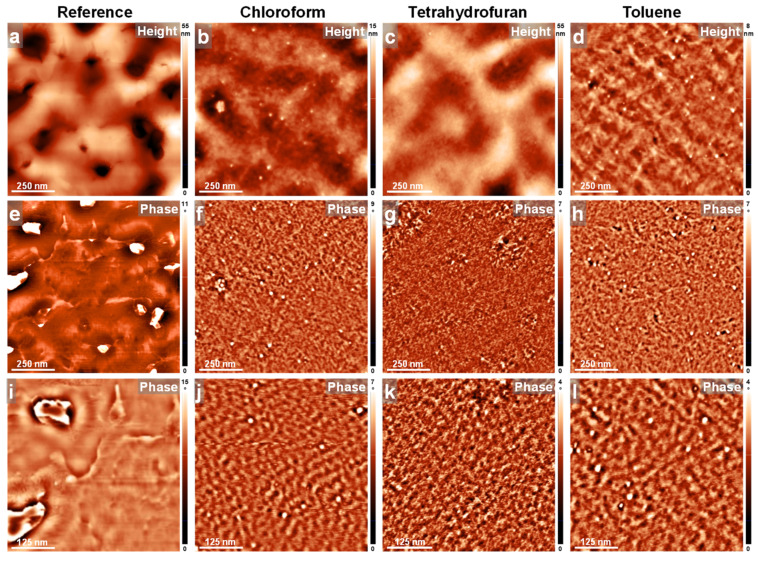
AFM height (**a**–**d**) and phase (**e**–**l**) micrographs depicting the surface morphology of PFO_63k_ thin films before (**a**,**e**,**i**) and after their exposure via the C-SVA method to chloroform (**b**,**f**,**j**), tetrahydrofuran (**c**,**g**,**k**) and toluene (**d**,**h**,**l**) vapors.

**Figure 7:**
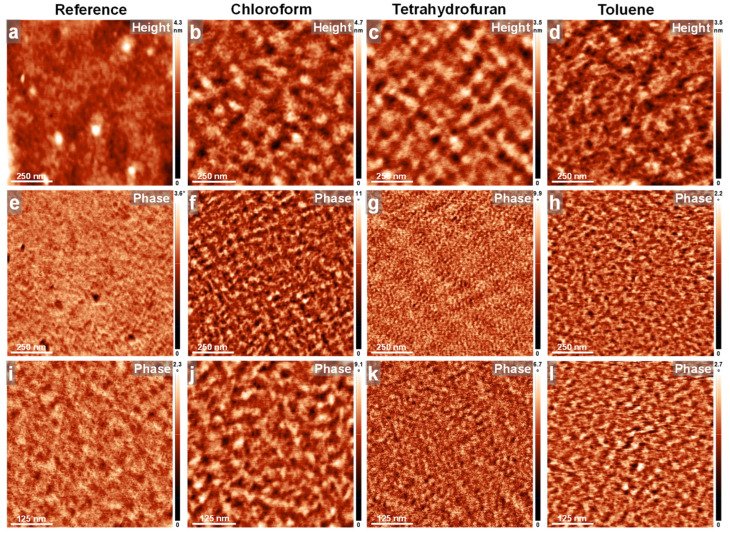
AFM height (**a**–**d**) and phase (**e**–**l**) micrographs depicting the surface morphology of PFO_14k_ thin films before (**a**,**e**,**i**) and after their exposure via the C-SVA method to chloroform (**b**,**f**,**j**), tetrahydrofuran (**c**,**g**,**k**) and toluene (**d**,**h**,**l**) vapors.

As in the case of the PFO_105k_ system, we further conducted AFM measurements on all PFO_63k_ and PFO_14k_ films before and after their exposure to the three types of solvent vapors via the C-SVA method. These results are summarized in Figure 6 and Figure 7. As shown in Figure 6, the reference spin-cast PFO_63k_ film displayed again a microstructure composed of a porous-like appearance (Figure 6a), but with pores being smaller and less deep as compared to those observed in the case of PFO_105k_. As demonstrated by the AFM phase contrast (Figure 6e), pores were surrounded by highly amorphous structures. Again, there were almost no signs of other features of molecular dimensions when zooming-in with the AFM (Figure 6i), pointing towards the idea of a rather amorphous morphology covering the surface of the spin-cast PFO_63k_ film, and in accordance with the absorption measurements (note that the absorption spectrum was found to be dominated by the large glassy/amorphous peak located at 397 nm [33,51,83]). In comparison, the PFO_63k_ films that were exposed to chloroform, tetrahydrofuran and toluene vapors via the C-SVA method displayed much fewer and less deep pores on the surfaces (see Figure 6b–d and Figure S11 in the SI). The average depths of the pores found in the reference unexposed PFO_63k_ film and in PFO_63k_ films exposed to chloroform, tetrahydrofuran and toluene vapors were measured to be around 60 ± 15 nm, 25 ± 12 nm, 40 ± 14 nm and 30 ± 14 nm, respectively. As revealed by the AFM phase contrast images presented in Figure 6f–h, as well as in Figure S11j–l in the SI, these pores were again filled with and surrounded by rather crystalline structures. Furthermore, when zooming-in with the AFM (Figure 6j–l), the surfaces appeared to be covered with substructures resembling lamellar features and displaying an average lateral width of about 11 ± 2 nm in the case of PFO_63k_ films exposed to tetrahydrofuran vapors, or a width about twice as much in the case of PFO_63k_ films exposed to chloroform and toluene vapors. Therefore, while all crystalline morphologies resembled the *β*-phase conformations [51,88,89] and were in good agreement with the existence of a smaller absorption peak at 431 nm generated by the crystalline phase [33,50,51,83,85,86,87] (Figure S10 in the SI), we do not exclude the possibility that PFO_63k_ chains exposed to tetrahydrofuran vapors experienced more folding, as opposed to those exposed to chloroform and toluene vapors. Interestingly, while a greater degree of chain folding would explain the lower average widths of the lamellar features observed in the PFO_63k_ film exposed to tetrahydrofuran vapors (Figure 6k), it does not necessarily mean that these chains are less extended between their folded extremities. This tentative statement could be supported by the highest increase in PL (258%) displayed by this film.

Similarly, the corresponding AFM data recorded for PFO_14k_ thin films further support the transformation of a predominant amorphous morphology towards a more structured one when PFO_14k_ films experienced exposure to various solvent vapors via the C-SVA method (Figure 7). Compared to rather porous surface morphologies previously observed in the longer PFO_105k_ and PFO_63k_ chains, the PFO_14k_ chains generated rather homogenous surfaces, with very few pores visible only on larger areas, as can be seen in Figure S12 in the SI (note that the average depths of the pores found in the reference unexposed PFO_14k_ film and in PFO_14k_ films exposed to chloroform, tetrahydrofuran and toluene vapors were measured to be around 90 ± 25 nm, 55 ± 15 nm, 32 ± 12 nm and 60 ± 25 nm, respectively). On smaller length scales, the AFM measurements conducted on PFO_14k_ films, before and after their exposure to the three types of solvent vapors, have revealed a surface microstructure composed of either fine, shapeless features corresponding to the unprocessed PFO_14k_ film (Figure 7a,e,i), or more ordered lamella-resembling features corresponding to the PFO_14k_ films C-SVA-processed in chloroform (Figure 7b,f,j), tetrahydrofuran (Figure 7c,g,k) and toluene (Figure 7d,h,l), respectively. As demonstrated by the AFM phase contrast images in Figure 7e,i, the surface of the PFO_14k_ film was already less amorphous than that of its longer-chain analogues, even before the C-SVA processing. It most probably already contained a higher fraction of *β*-phase conformations formed right after the spin-casting process (this is likely to happen for polymer chains of relatively low molecular weight), possibly explaining the weaker PL enhancement observed in PFO_14k_ films upon their further processing via the C-SVA method. Moreover, by analyzing several AFM cross-sectional profiles for all C-SVA-processed films (Figure 7j–l), the lateral dimension of the lamellar features resembling *β*-phase arrangements was deduced to be 19 ± 4 nm. Nonetheless, such ordered features were more visible in the case of the PFO_14k_ film that was processed in tetrahydrofuran vapors (Figure 7k).

To further demonstrate the alteration of the glassy phase and its partial transformation into additional *β*-phase molecular arrangements upon the C-SVA processing of PFO_63k_ and PFO_14k_ films, we have gathered additional Raman data (see Figure S13 and Figure S14 in the SI, respectively). As can be seen in Figure S13, the 1000–1650 cm^−1^ spectral region representing the fluorene unit motion in the main chain [90] and related to the vibrations of fluorene backbone displayed again a visible peak at 1604 cm^−1^, with a small shoulder at 1580 cm^−1^ (inset in Figure S13a), indicating a strong presence of *β*-phase conformations [52]. Moreover, upon the C-SVA processing of PFO_63k_ films, the intensity of Raman peaks in the 1100–1400 cm^−1^ spectral region (including the peaks at 1134 cm^−1^, 1174 cm^−1^, 1188 cm^−1^, 1221 cm^−1^ and 1254–1159 cm^−1^), attributed to C–C stretching modes between the phenylene rings and C–H in-plane bending modes [90], increased (see the region delimited by the dotted box in Figure S13a; see also Figures S13b and S15 in the SI). This was due to the increase in the fraction of *β*-phase [52] and PFO_63k_ chain planarization [93,94]. Again, the two distinct bands at 1259 cm^−1^ and 1280 cm^−1^ clearly intensified upon the C-SVA processing of PFO_63k_ thin films in all three types of solvent vapors (Figure S13b), further indicating an increase in the planarity of the PFO_63k_ main chains [52] and thus demonstrating the transformation of a certain fraction of less planar primal chain conformations into more planar *β*-phase conformations, in agreement with our absorption, PL and AFM measurements.

Similar Raman behavior was also observed for the shortest PFO_14k_ chains. The obtained Raman results have further demonstrated the partial transformation, upon the C-SVA processing of PFO_14k_ films in three different solvent vapors, of the glassy phase into additional *β*-phase conformations (Figure S14 in the SI). In this case, the 1000–1650 cm^−1^ spectral region exhibited again a visible peak at 1604 cm^−1^ along with a small shoulder at 1580 cm^−1^ (inset in Figure S14a), indicating the existence of *β*-phase arrangements [52]. Moreover, the intensity of Raman peaks from the 1100–1400 cm^−1^ spectral region (including the peaks at 1117 cm^−1^, 1133 cm^−1^, 1170 cm^−1^, 1221 cm^−1^ and 1233 cm^−1^), attributed to C–C stretching modes between the phenylene rings and C–H in-plane bending modes [90], also increased (see Figures S14b and S16 in the SI), just like in the case of PFO_105k_ and PFO_63k_ films. This was due to the increase in the *β*-phase fraction [52]. Furthermore, the two bands at 1255 cm^−1^ and 1279 cm^−1^ intensified again upon the C-SVA processing of PFO_14k_ thin films in all three types of solvent vapors (Figure S14b), and led to more planarized PFO_14k_ chain conformations [52], thus further demonstrating the increase in the *β*-phase fraction. At the end, we would like to point out that no visible spectral differences were identified when comparing Raman data with respect to the molecular mass of PFO_105k_, PFO_63k_ and PFO_14k_ systems, neither before nor after their C-SVA processing in chloroform vapors (compare Figure S17 with Figure S18 in the SI).

## 4. Conclusions

The fabrication of thin films of conjugated polymers exhibiting an optimized microstructure is of paramount importance when designing and developing, for instance, novel and better optoelectronic applications. It is well accepted nowadays that such films can be fabricated by the employment of highly efficient polymer processing methods. Here, we have studied thin PFO films of three different molecular weights before and after their exposure to various solvent vapors via C-SVA processing. We have found that after such processing, all PFO films exhibited an important enhancement in their emission properties, with the longest PFO_105k_ chains emitting light more efficiently and demonstrating enlargements in PL intensity of up to 270%. In comparison, weaker PL enlargements of 258% and 240% was obtained for thin films of PFO_63k_ and PFO_14k_, respectively. By considering additional UV-Vis, AFM and Raman measurements, we have assigned this enlargement of PL to the increase, upon C-SVA processing, of the fraction composed of more extended and more planar *β*-phase conformations. These latter conformations showed longer conjugation lengths and most probably favored the delocalization of π electrons along the conjugated backbones. The enhancement of the emission properties of thin films of conjugated polymers presented in this study could be further taken into consideration when developing novel light-emitting diodes and various state-of-the-art displays of better efficiency and eventually adjustable colors.

## Figures and Tables

**Table 1:** Comparison of PL data inferred from the emission spectra recorded for PFO_105k_, PFO_63k_ and PFO_14k_ films before and after their C-SVA processing in three different solvent vapors.

Material	Solvent	Abs. λ (nm)	PL λ (nm)	Area Ratio ^a^	SW λ_0-0_ Peak Ratio ^b^	LW λ_0-1_ Peak Ratio ^c^
PFO_105k_	tetrahydrofuran	-	λ_0-0_ = 440	1.824	2.705	2.475
		-	λ_0-1_ = 467			
		-	λ_fd_ = 500			
	toluene	λ_g_ = 397	λ_0-0_ = 440	1.747	2.501	2.325
		λ_β_ = 431	λ_0-1_ = 467			
		-	λ_fd_ = 500			
	chloroform	-	λ_0-0_ = 440	1.706	2.474	2.319
		-	λ_0-1_ = 467			
		-	λ_fd_ = 500			
	reference	λ_g_ = 391	λ_0-0_ = 426	-	-	-
		-	λ_0-1_ = 445			
PFO_63k_	tetrahydrofuran	-	λ_0-0_ = 439	1.659	2.581	2.033
		-	λ_0-1_ = 466			
		-	λ_fd_ = 500			
	toluene	λ_g_ = 397	λ_0-0_ = 439	1.676	2.533	2.066
		λ_β_ = 431	λ_0-1_ = 466			
		-	λ_fd_ = 500			
	chloroform	-	λ_0-0_ = 440	1.612	2.402	2.094
		-	λ_0-1_ = 467			
		-	λ_fd_ = 500			
	reference	λ_g_ = 392	λ_0-0_ = 424	-	-	-
		-	λ_0-1_ = 445			
PFO_14k_	tetrahydrofuran	-	λ_0-0_ = 440	1.886	2.068	2.504
		-	λ_0-1_ = 467			
		-	λ_fd_ = 500			
	toluene	λ_g_ = 398	λ_0-0_ = 439	1.952	2.404	2.359
		λ_β_ = 431	λ_0-1_ = 466			
		-	λ_fd_ = 500			
	chloroform	-	λ_0-0_ = 440	2.179	2.291	2.664
		-	λ_0-1_ = 467			
		-	λ_fd_ = 500			
	reference	λ_g_ = 393	λ_0-0_ = 426	-	-	-
		-	λ_0-1_ = 444			

Abs. = absorption; λ_g_ = wavelength of the absorption peak representing the glassy phase; λ_β_ = wavelength of the absorption peak representing the *β*-phase; λ_fd_= wavelength of the absorption peak attributed inclusively to fluorenone defects; ^a^ = obtained by dividing the total area under the PL curves corresponding to the C-SVA-processed and unprocessed films; ^b^ = obtained by dividing the maximum PL intensity of SW peaks corresponding to the C-SVA-processed and unprocessed films; ^c^ = obtained by dividing the maximum PL intensity of longer-wavelength (LW) peaks corresponding to the C-SVA-processed and unprocessed films.

## Data Availability

The data are contained within this article and Supplementary Materials.

## Supplementary Materials

The following supporting information can be downloaded at: https://www.mdpi.com/article/10.3390/polym16162278/s1, Figure S1: (a–d) Digital images of PFO_105k_ films before (a) and after their processing via C-SVA method in tetrahydrofuran (b), chloroform (c) and toluene (d) vapors, respectively. (e–f) Digital images of two unprocessed and C-SVA processed (in toluene) PFO_105k_ films under a UV-lamp; Figure S2: Normalized absorption spectra of a PFO_105k_ film before (dotted line) and after (solid line) its exposure to toluene vapors via the C-SVA method. Dotted vertical grey lines are for guiding the eye only; Figure S3: AFM height (a–h) and phase (i–l) micrographs depicting the morphology of PFO_105k_ thin films before (a,e,i) and after their exposure to chloroform (b,f,j), tetrahydrofuran (c,g,k) and toluene (d,h,l) vapors; Figure S4: Raman spectra recorded for a spin cast PFO_105k_ film before (black) and after its processing via the C-SVA method in tetrahydrofuran (blue), chloroform (red) and toluene (olive) vapors, respectively. These spectra depict the zoomed-in spectral interval of 1000–1500 cm^−1^ and are emphasizing the changes of various peaks induced upon the C-SVA processing. Grey vertical dotted arrows are for guiding the eye only; Figure S5: PL (a) and normalized PL (b) spectra of PFO_63k_ films before (black) and after their processing via the C-SVA method in tetrahydrofuran (blue), chloroform (red) and toluene (olive) vapors, along with their corresponding digital images (c–f), respectively. All PL spectra were acquired using an excitation wavelength of 390 nm; Figure S6: PL (a) and normalized PL (b) spectra of PFO_14k_ films before (black) and after their processing via the C-SVA method in tetrahydrofuran (blue), chloroform (red) and toluene (olive) vapors, along with their corresponding digital images (c–e), respectively. All PL spectra were acquired using an excitation wavelength of 390 nm. Figure S7: Normalized absorption spectra of a PFO_63k_ film before (dotted line) and after (solid line) its exposure to toluene vapors via the C-SVA method. Dotted vertical grey lines are for guiding the eye only; Figure S8: Normalized absorption spectra of a PFO_14k_ film before (dotted line) and after (solid line) its exposure to toluene vapors via the C-SVA method. Dotted vertical grey lines are for guiding the eye only; Figure S9: (a–d) Digital images of PFO_63k_ films before (a) and after their processing via the C-SVA method in tetrahydrofuran (b), chloroform (c) and toluene (d) vapors, respectively. (e–f) Digital images of two unprocessed and C-SVA processed (in toluene) PFO_63k_ films under a UV-lamp; Figure S10: Optical images of PFO_14k_ films before (a) and after their processing via the C-SVA method in tetrahydrofuran (b), chloroform (c) and toluene (d) vapors; Figure S11: AFM height (a–h) and phase (i–l) micrographs depicting the morphology of PFO_63k_ thin films before (a,e,i) and after their exposure to chloroform (b,f,j), tetrahydrofuran (c,g,k) and toluene (d,h,l) vapors; Figure S12: AFM height (a–h) and phase (i–l) micrographs depicting the morphology of PFO_14k_ thin films before (a,e,i) and after their exposure to chloroform (b,f,j), tetrahydrofuran (c,g,k) and toluene (d,h,l) vapors; Figure S13: (a) Raman spectra recorded for an as spin cast film of PFO_63k_ system before (black) and after its processing via the C-SVA method in tetrahydrofuran (blue), chloroform (red) and toluene (olive) vapors, respectively. The inset depicts a zoom-in of the main peak located at around 1604 cm^−1^. (b) Same vertically translated Raman spectra zoomed-in in the 1000–1500 cm^−1^ spectral interval, as indicated by the dotted shape in (a), emphasizing the changes of various peaks induced upon the C-SVA processing. Grey vertical dotted lines/arrows in (b) are for guiding the eye only; Figure S14: (a) Raman spectra recorded for an as spin cast film of PFO_14k_ system before (black) and after its processing via the C-SVA method in tetrahydrofuran (blue), chloroform (red) and toluene (olive) vapors, respectively. The inset depicts a zoom-in of the main peak located at around 1604 cm^−1^. (b) Same vertically translated Raman spectra zoomed-in in the 1000–1500 cm^−1^ interval, as indicated by the dotted shape in (a), emphasizing the changes of various peaks induced upon the C-SVA processing. Grey vertical dotted lines/arrows in (b) are for guiding the eye only; Figure S15: Raman spectra recorded for a spin-cast PFO_63k_ film before (black) and after its processing via the C-SVA method in tetrahydrofuran (blue), chloroform (red) and toluene (olive) vapors, respectively. These spectra depict the zoomed-in spectral interval of 1000–1500 cm^−1^ and are emphasizing the changes of various peaks induced upon the C-SVA processing. Grey vertical dotted lines/arrows are for guiding the eye only; Figure S16: Raman spectra recorded for a spin-cast PFO_14k_ film before (black) and after its processing via the C-SVA method in tetrahydrofuran (blue), chloroform (red) and toluene (olive) vapors, respectively. These spectra depict the zoomed-in spectral interval of 1000–1500 cm^−1^ and are emphasizing the changes of various peaks induced upon the C-SVA processing. Grey dotted lines/arrows are for guiding the eye only; Figure S17: Raman spectra recorded for as spin-cast films of PFO_14k_ (blue), PFO_63k_ (red) and PFO_105k_ (black) systems before their processing via the C-SVA method. These spectra are depicting the zoomed-in 1000–1500 cm^−1^ spectral interval. As we can see, no visible spectral differences were identified with respect to the molecular mass of PFO polymers; Figure S18: Raman spectra recorded for as spin-cast films of PFO_14k_ (blue), PFO_63k_ (red) and PFO_105k_ (black) systems that were processed via the C-SVA method in chloroform vapors. These spectra are depicting the zoomed-in 1000–1500 cm^−1^ spectral interval. As we can see, no visible spectral differences were identified with respect to the molecular mass of PFO polymers when processed via the C-SVA method.
