# Supplementary material for: Large Enhancement of Photoluminescence Obtained in Thin Polyfluorene Films of Optimized Microstructure
Source: Polymers (Basel). 2024 Aug 11;16(16):2278. doi: 10.3390/polym16162278 (PMC11359287; doi:10.3390/polym16162278)
Supplement: Supplementary file 1 [file polymers-16-02278-s001.zip › polymers-3134765-supplementary.pdf]

Supporting information for:

Large Enhancement of Photoluminescence Obtained in Thin Polyfluorene Films of Optimized Microstructure

Otto Todor-Boer[1], Cosmin Farcău[2,3], Ioan Botiz[3,4,*]

[1]*Research Institute for Analytical Instrumentation Subsidiary, National Institute for Research and Development of Optoelectronics Bucharest INOE 2000, 67 Donath Street, 400293 Cluj-Napoca, Romania; otto.todor@icia.ro*

[2]*National Institute for Research and Development of Isotopic and Molecular Technologies INCDTIM, 67-103 Donath Street, 400293 Cluj-Napoca, Romania; cosmin.farcau@itim-cj.ro*

[3]*Interdisciplinary Research Institute on Bio-Nano-Sciences, Babeș-Bolyai University, 400271 Cluj-Napoca, Romania*

[4]*Department of Physics of Condensed Matter and Advanced Technologies, Faculty of Physics, Babeș-Bolyai University, 400084 Cluj-Napoca, Romania*

**Correspondence: ioan.botiz@ubbcluj.ro*

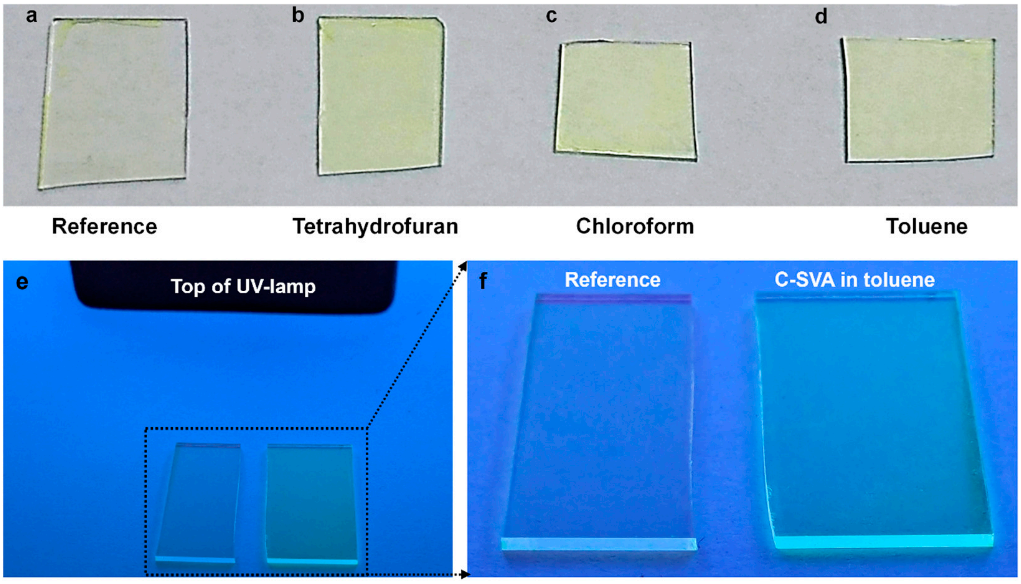

Figure S1. (a-d) Digital images of PFO_{105k} films before (a) and after their processing via C-SVA method in tetrahydrofuran (b), chloroform (c) and toluene (d) vapors, respectively. (e-f) Digital images of two unprocessed and C-SVA processed (in toluene) PFO_{105k} films under a UV-lamp.

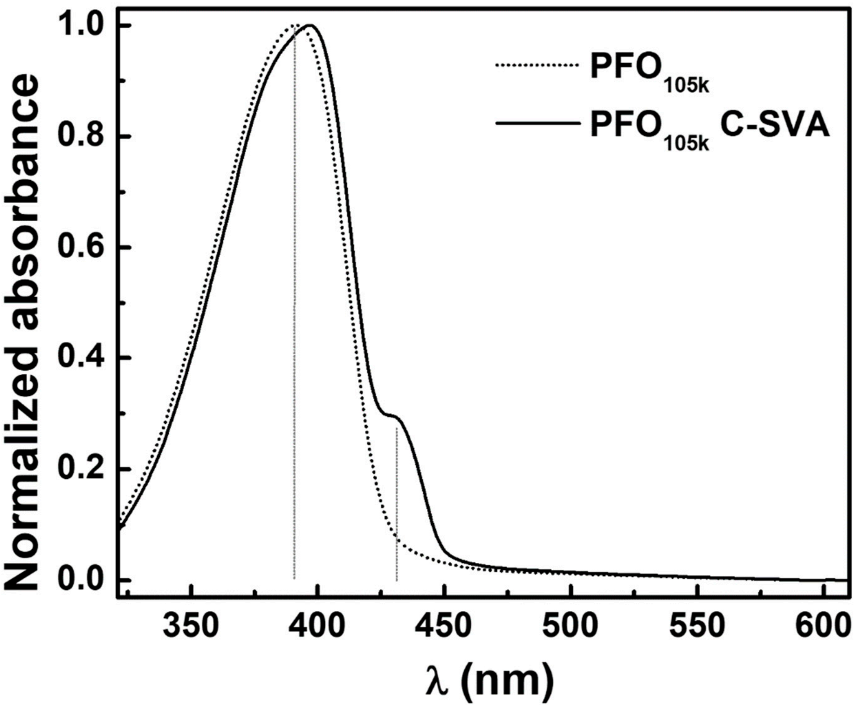

Figure S2. Normalized absorption spectra of a PFO_{105k} film before (dotted line) and after (solid line) its exposure to toluene vapors via the C-SVA method. Dotted vertical grey lines are for guiding the eye only.

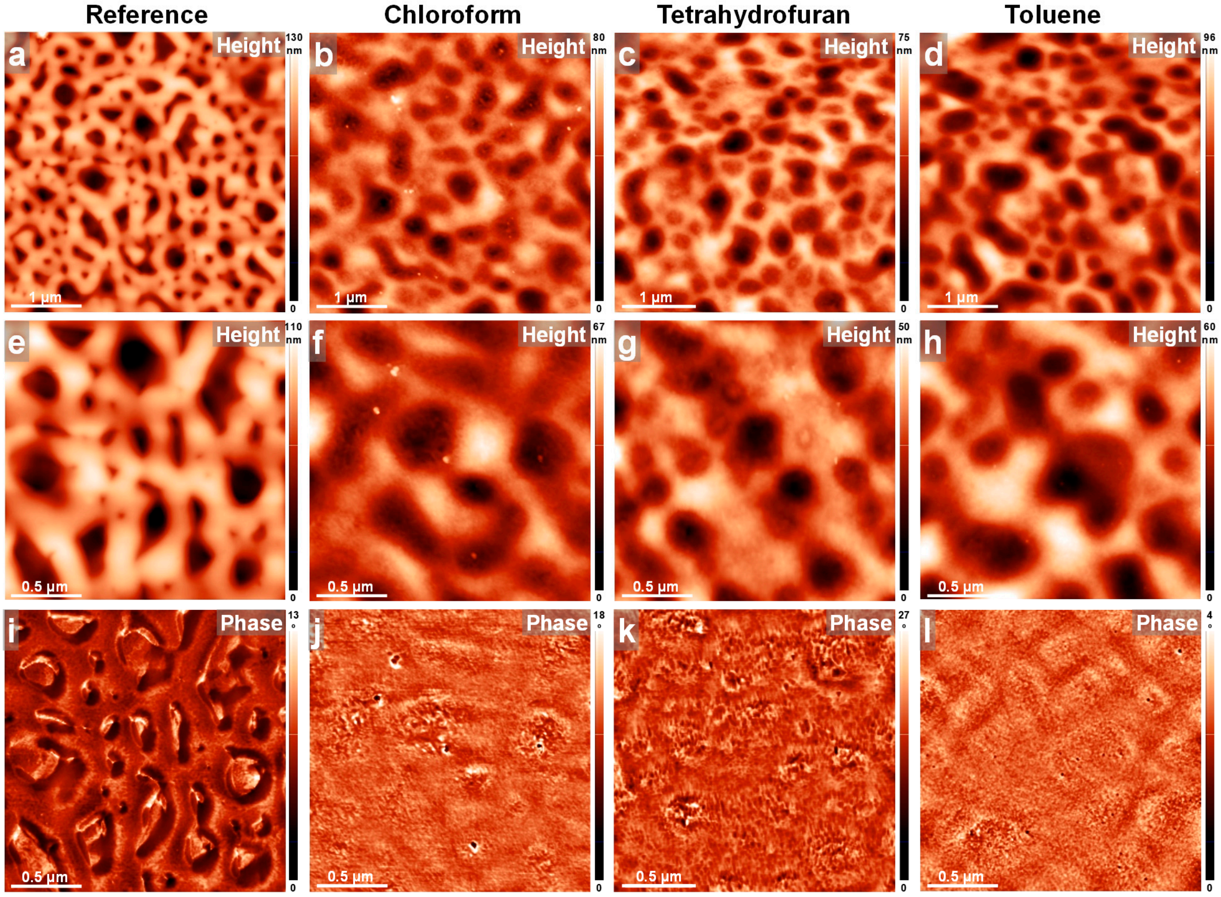

Figure S3. AFM height (a-h) and phase (i-l) micrographs depicting the morphology of PFO_{105k} thin films before (a, e, i) and after their exposure to chloroform (b, f, j), tetrahydrofuran (c, g, k) and toluene (d, h, l) vapors.

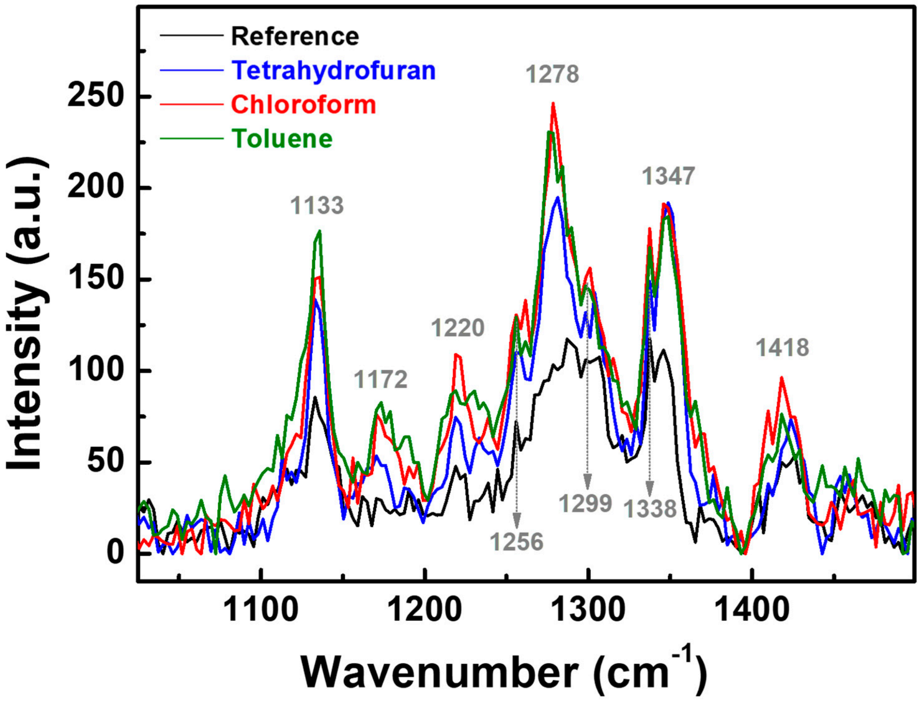

Figure S4. Raman spectra recorded for a spin cast PFO_{105k} film before (black) and after its processing via the C-SVA method in tetrahydrofuran (blue), chloroform (red) and toluene (olive) vapors, respectively. These spectra depict the zoomed-in spectral interval of 1000-1500 cm^{-1} and are emphasizing the changes of various peaks induced upon the C-SVA processing. Grey vertical dotted arrows are for guiding the eye only.

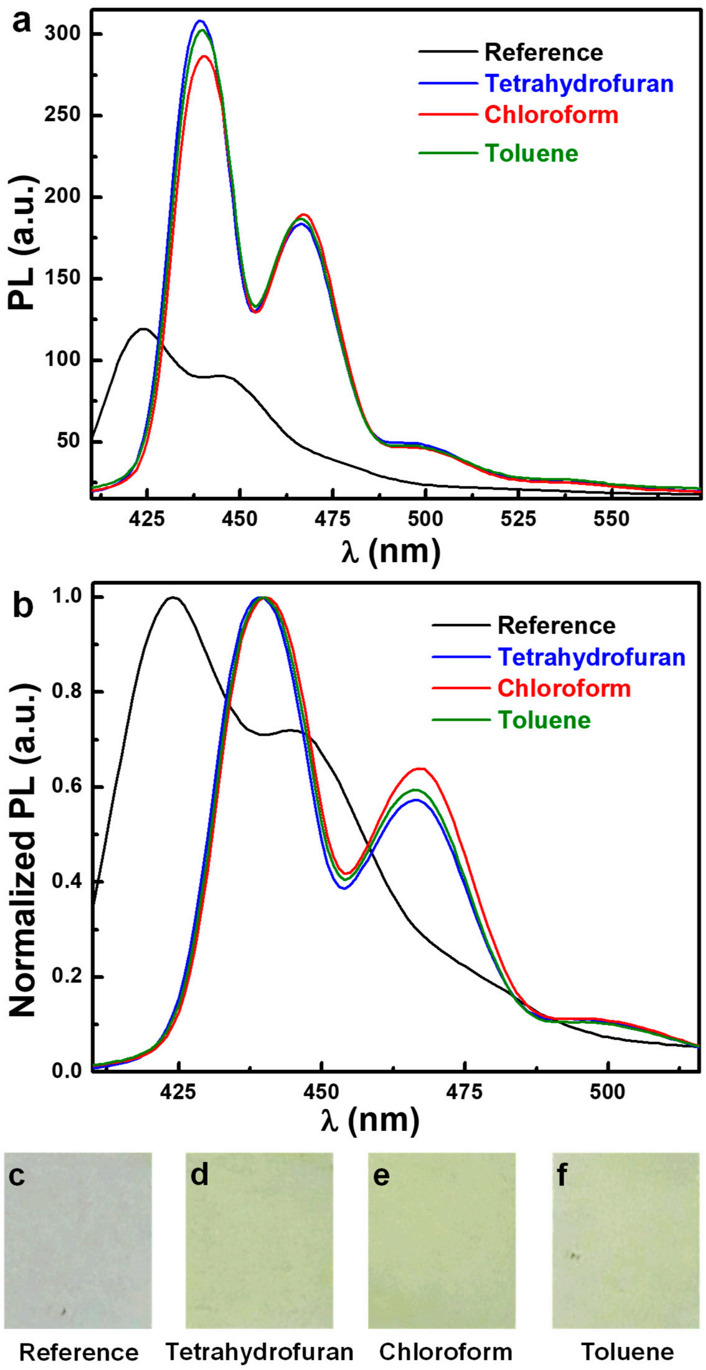

Figure S5. PL (a) and normalized PL (b) spectra of PFO_{63k} films before (black) and after their processing via the C-SVA method in tetrahydrofuran (blue), chloroform (red) and toluene (olive) vapors, along with their corresponding digital images (c-f), respectively. All PL spectra were acquired using an excitation wavelength of 390 nm.

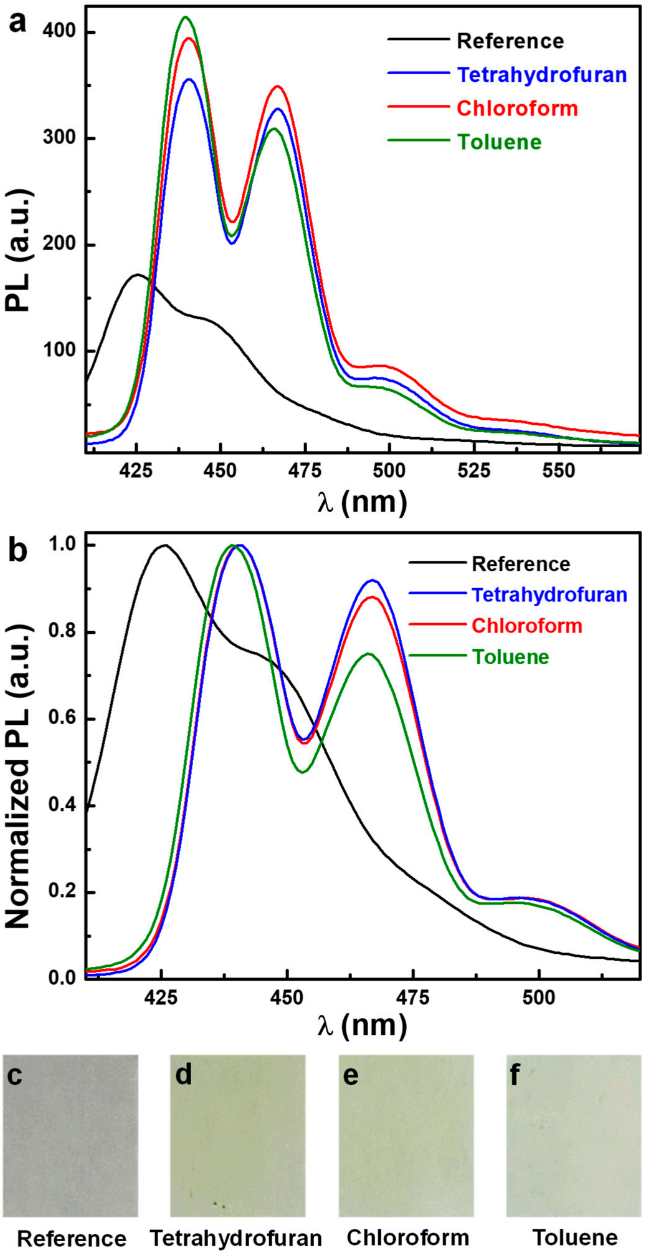

Figure S6. PL (a) and normalized PL (b) spectra of PFO_{14k} films before (black) and after their processing via the C-SVA method in tetrahydrofuran (blue), chloroform (red) and toluene (olive) vapors, along with their corresponding digital images (c-e), respectively. All PL spectra were acquired using an excitation wavelength of 390 nm.

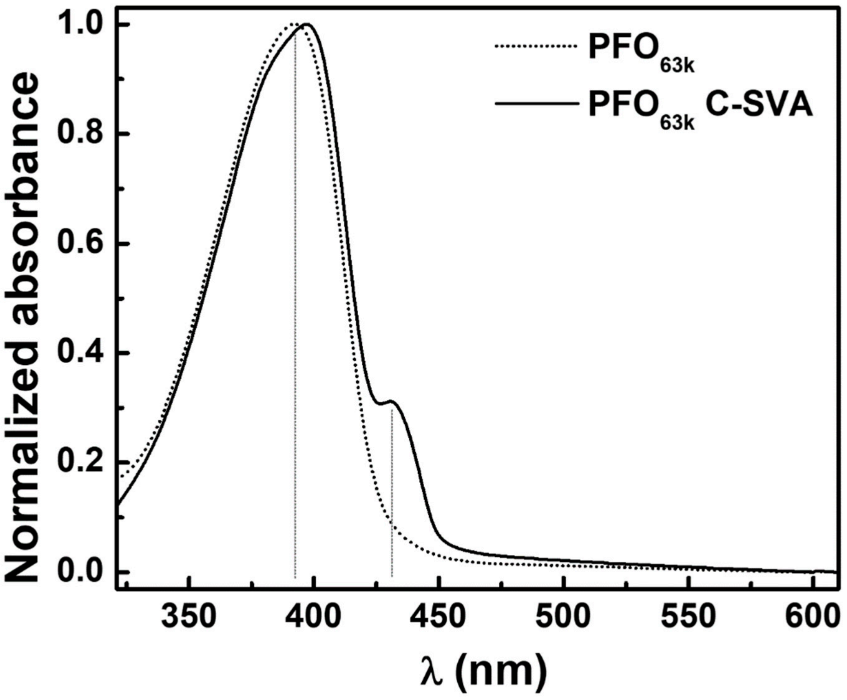

Figure S7. Normalized absorption spectra of a PFO_{63k} film before (dotted line) and after (solid line) its exposure to toluene vapors via the C-SVA method. Dotted vertical grey lines are for guiding the eye only.

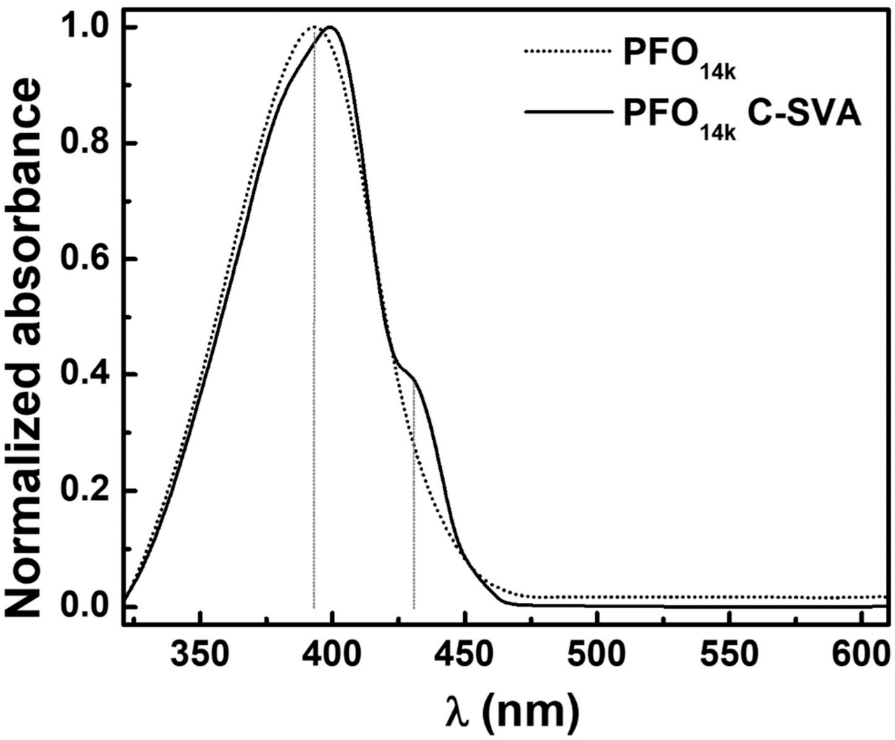

Figure S8. Normalized absorption spectra of a PFO_{14k} film before (dotted line) and after (solid line) its exposure to toluene vapors via the C-SVA method. Dotted vertical grey lines are for guiding the eye only.

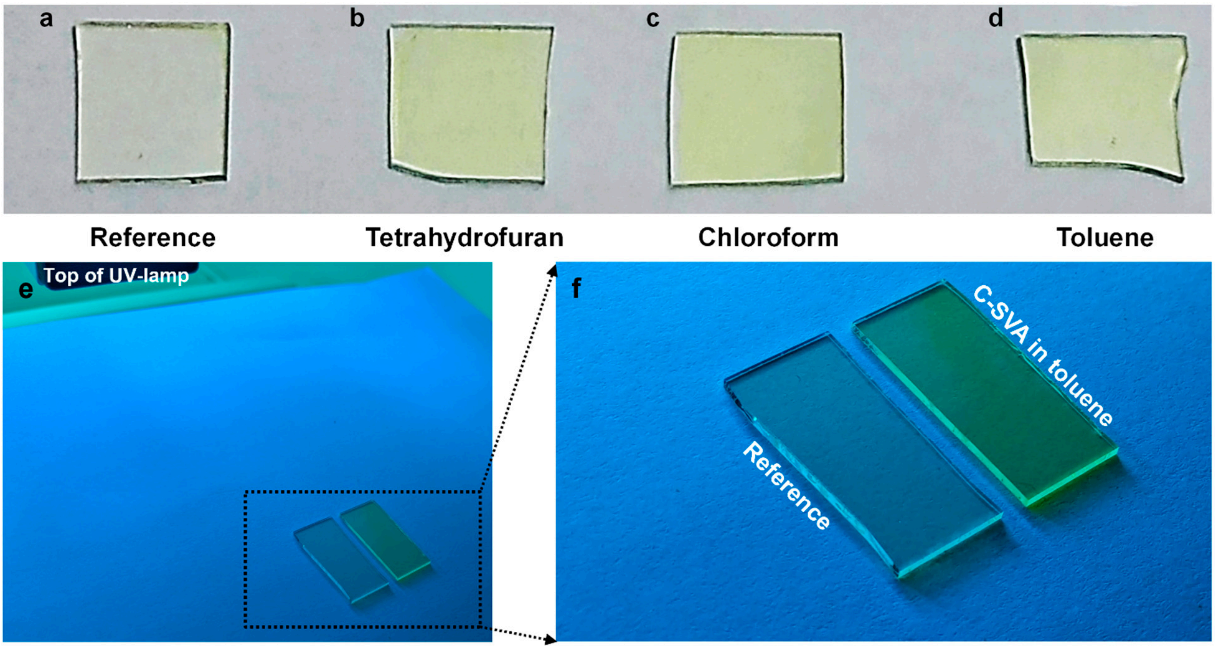

Figure S9. (a-d) Digital images of PFO_{63k} films before (a) and after their processing via the C-SVA method in tetrahydrofuran (b), chloroform (c) and toluene (d) vapors, respectively. (e-f) Digital images of two unprocessed and C-SVA processed (in toluene) PFO_{63k} films under a UV-lamp.

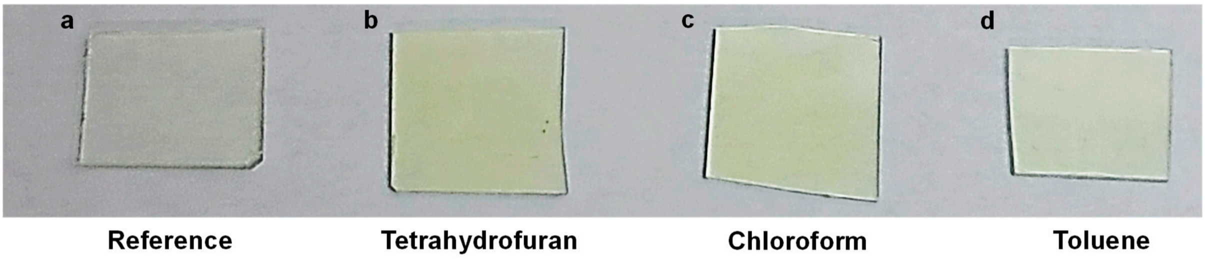

Figure S10. Optical images of PFO_{14k} films before (a) and after their processing via the C-SVA method in tetrahydrofuran (b), chloroform (c) and toluene (d) vapors.

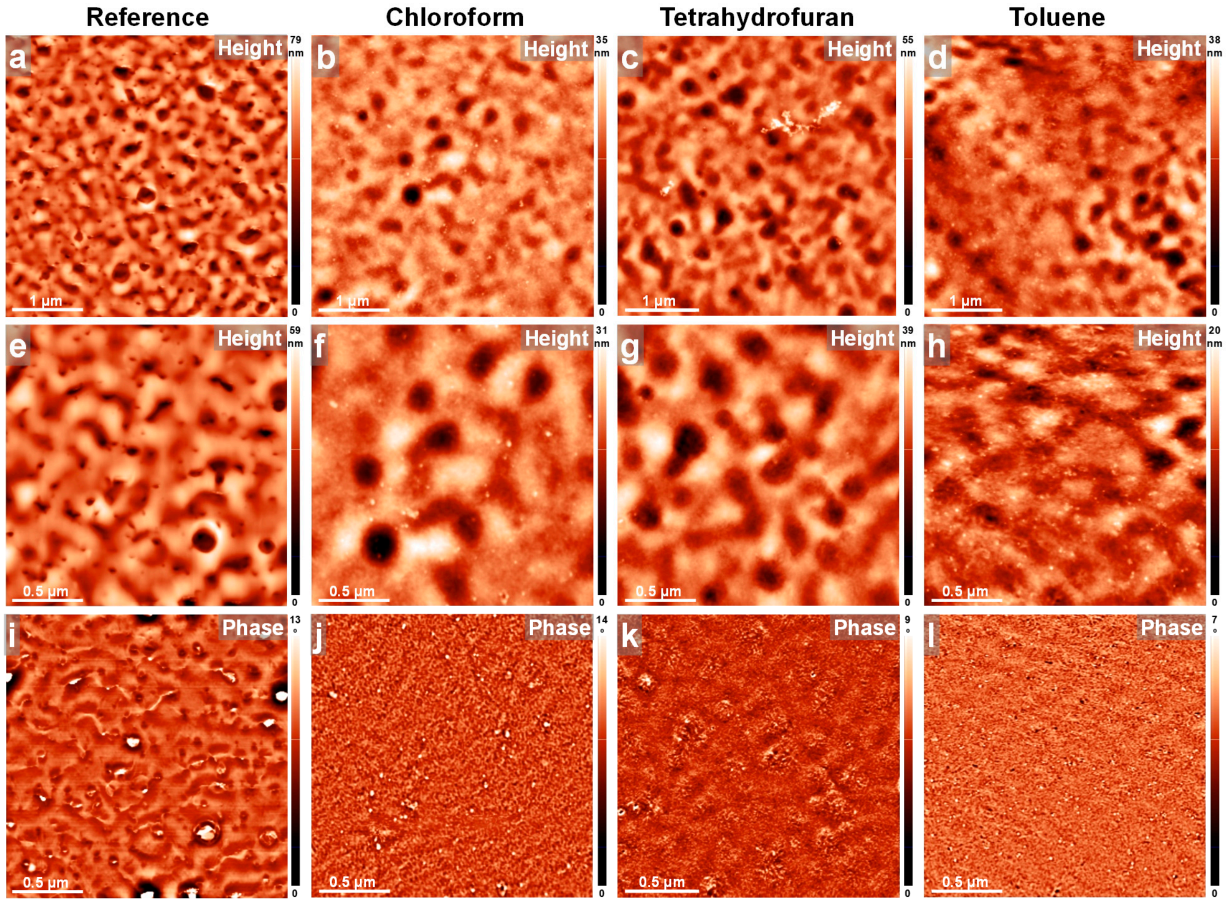

Figure S11. AFM height (a-h) and phase (i-l) micrographs depicting the morphology of PFO_{63k} thin films before (a, e, i) and after their exposure to chloroform (b, f, j), tetrahydrofuran (c, g, k) and toluene (d, h, l) vapors.

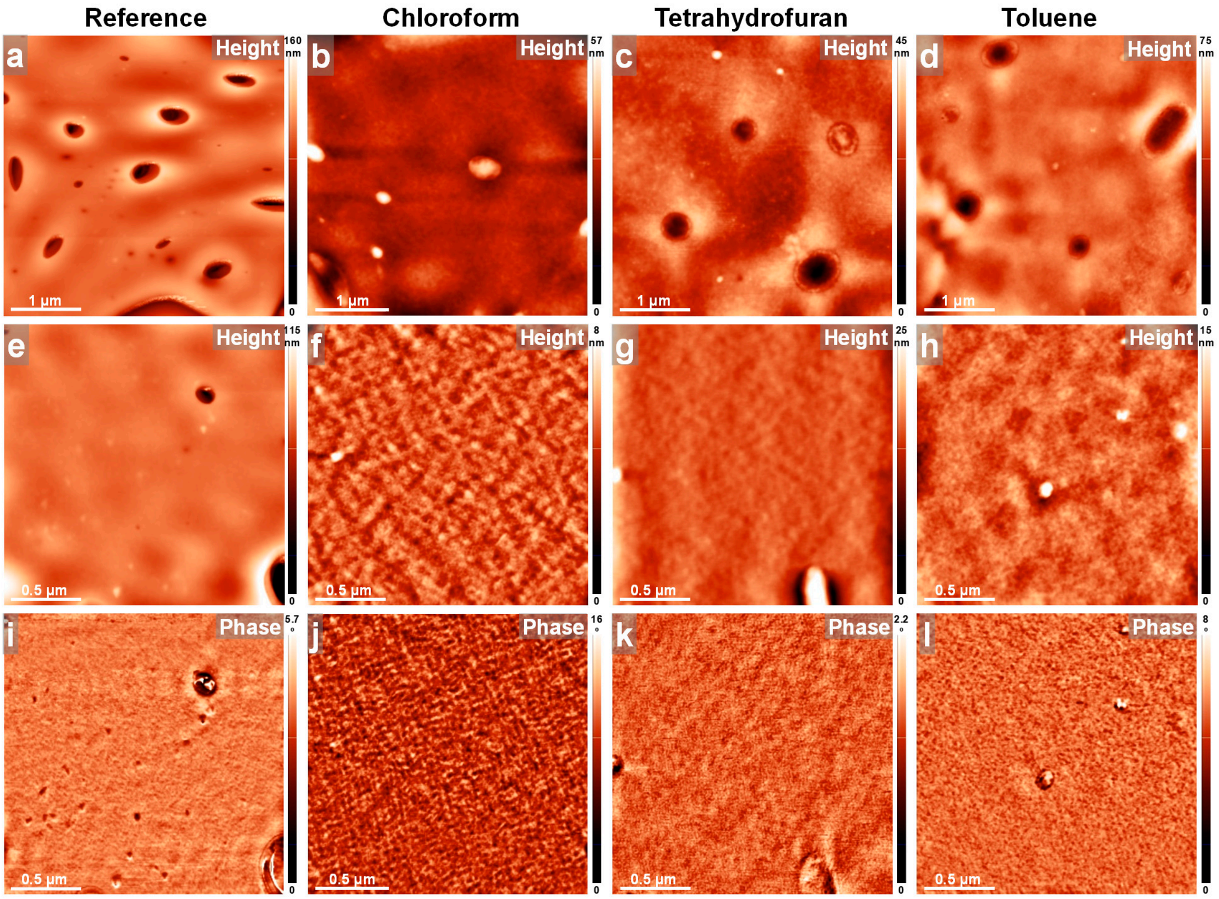

Figure S12. AFM height (a-h) and phase (i-l) micrographs depicting the morphology of PFO_{14k} thin films before (a, e, i) and after their exposure to chloroform (b, f, j), tetrahydrofuran (c, g, k) and toluene (d, h, l) vapors.

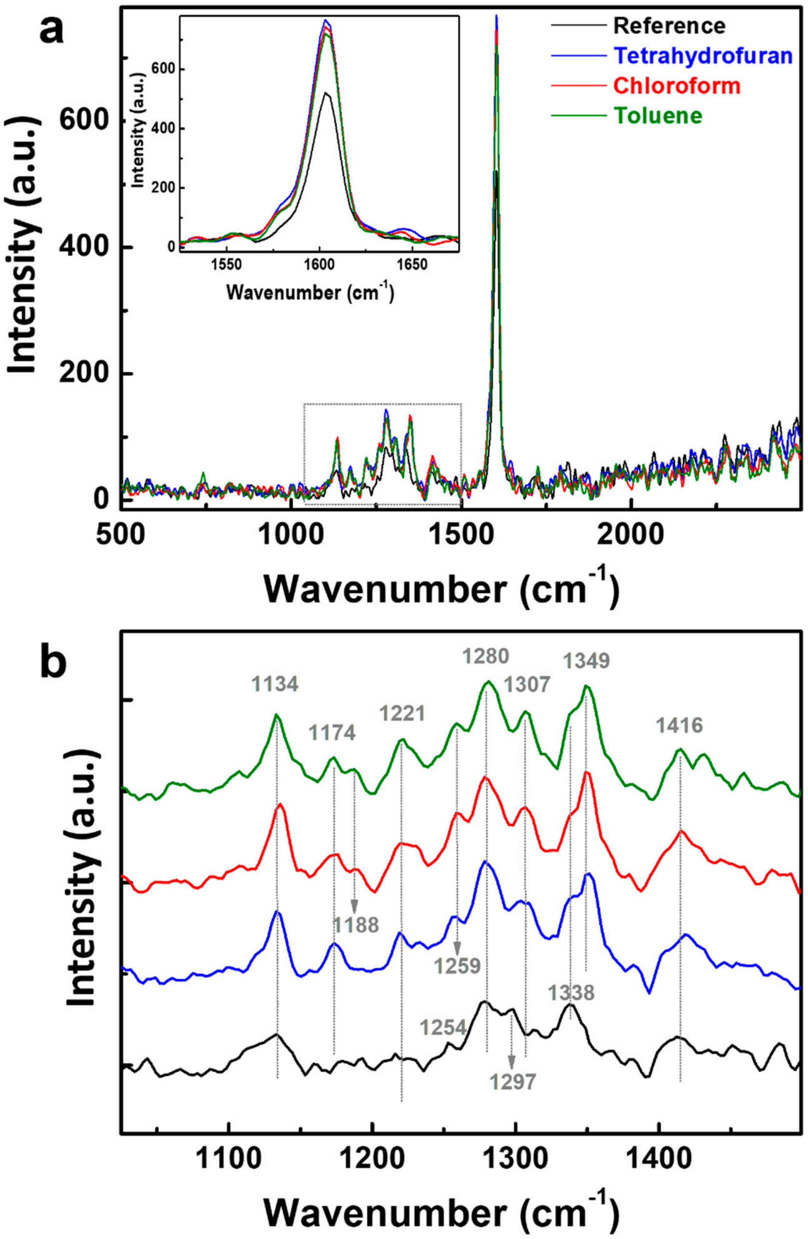

Figure S13. (a) Raman spectra recorded for an as spin cast film of PFO_{63k} system before (black) and after its processing via the C-SVA method in tetrahydrofuran (blue), chloroform (red) and toluene (olive) vapors, respectively. The inset depicts a zoom-in of the main peak located at around 1604 cm^{-1}. (b) Same vertically translated Raman spectra zoomed-in in the 1000-1500 cm^{-1} spectral interval, as indicated by the dotted shape in (a), emphasizing the changes of various peaks induced upon the C-SVA processing. Grey vertical dotted lines/arrows in (b) are for guiding the eye only.

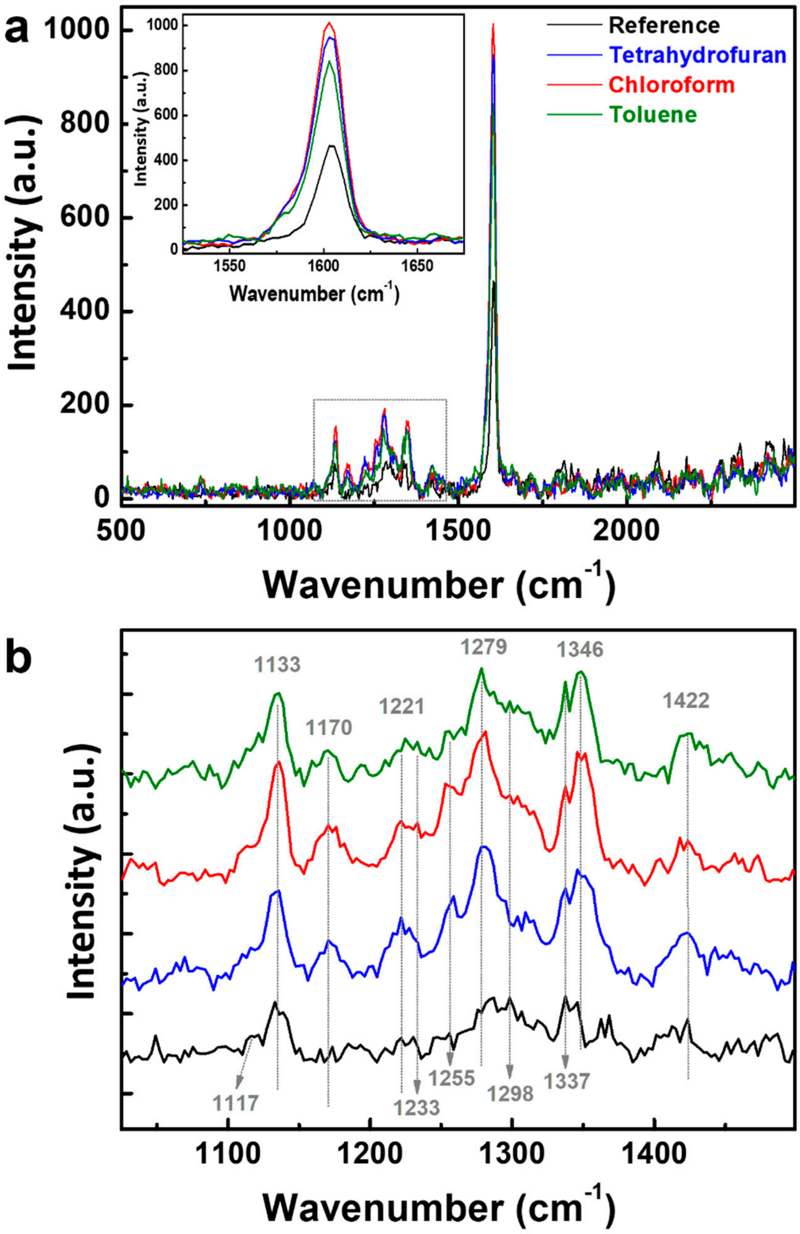

Figure S14. (a) Raman spectra recorded for an as spin cast film of PFO_{14k} system before (black) and after its processing via the C-SVA method in tetrahydrofuran (blue), chloroform (red) and toluene (olive) vapors, respectively. The inset depicts a zoom-in of the main peak located at around 1604 cm^{-1}. (b) Same vertically translated Raman spectra zoomed-in in the 1000-1500 cm^{-1} interval, as indicated by the dotted shape in (a), emphasizing the changes of various peaks induced upon the C-SVA processing. Grey vertical dotted lines/arrows in (b) are for guiding the eye only.

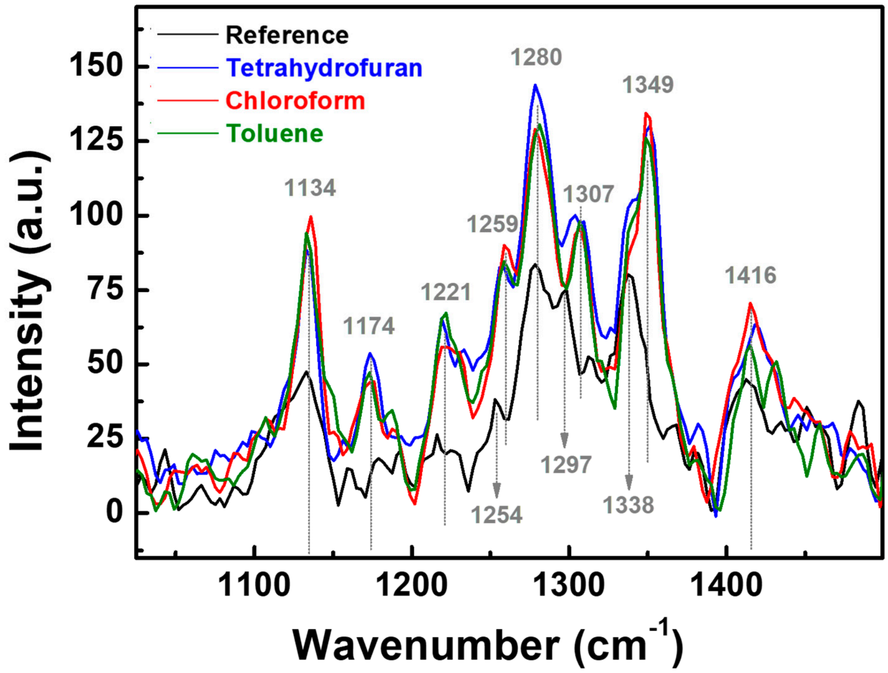

Figure S15. Raman spectra recorded for a spin-cast PFO_{63k} film before (black) and after its processing via the C-SVA method in tetrahydrofuran (blue), chloroform (red) and toluene (olive) vapors, respectively. These spectra depict the zoomed-in spectral interval of 1000-1500 cm^{-1} and are emphasizing the changes of various peaks induced upon the C-SVA processing. Grey vertical dotted lines/arrows are for guiding the eye only.

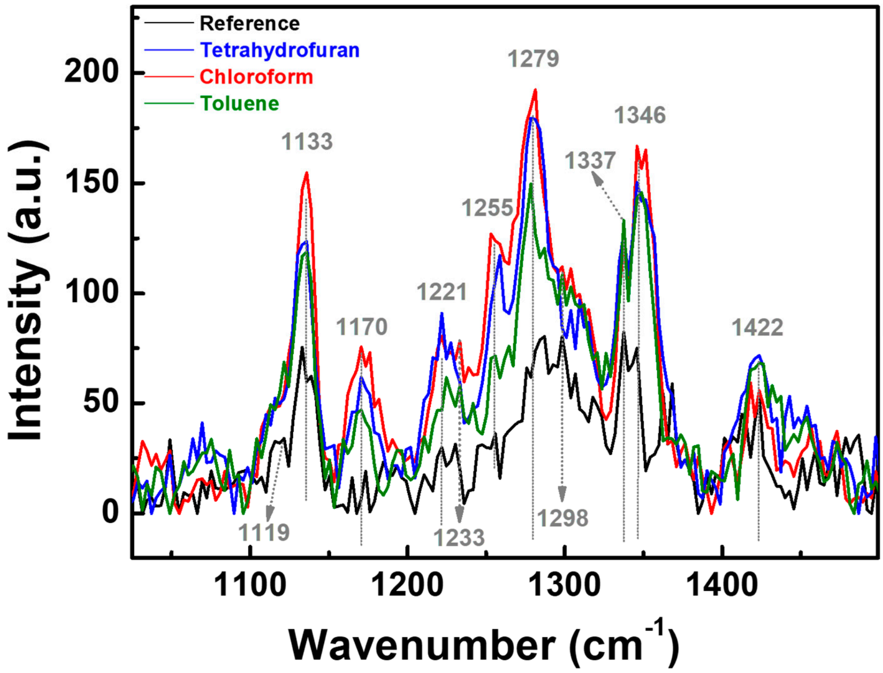

Figure S16. Raman spectra recorded for a spin-cast PFO_{14k} film before (black) and after its processing via the C-SVA method in tetrahydrofuran (blue), chloroform (red) and toluene (olive) vapors, respectively. These spectra depict the zoomed-in spectral interval of 1000-1500 cm^{-1} and are emphasizing the changes of various peaks induced upon the C-SVA processing. Grey dotted lines/arrows are for guiding the eye only.

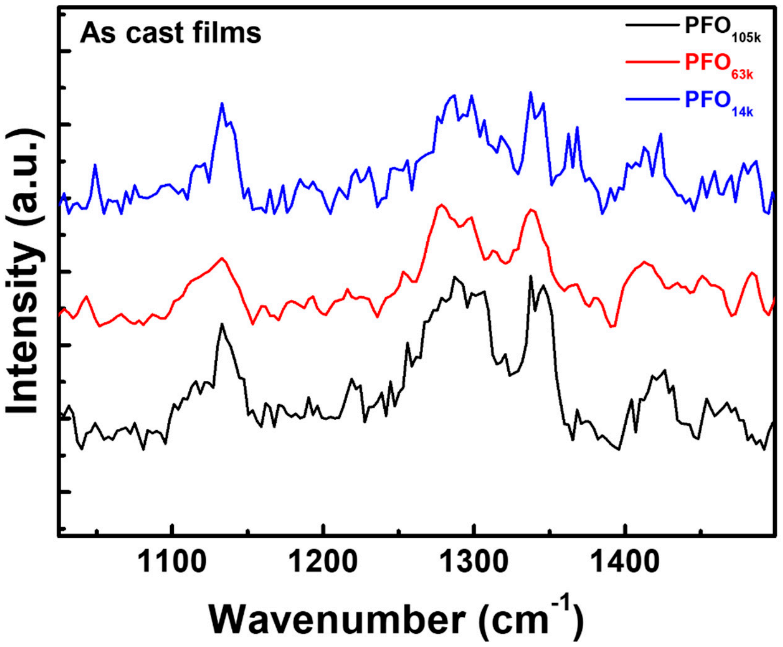

Figure S17. Raman spectra recorded for as spin-cast films of PFO_{14k} (blue), PFO_{63k} (red) and PFO_{105k} (black) systems before their processing via the C-SVA method. These spectra are depicting the zoomed-in 1000-1500 cm^{-1} spectral interval. As we can see, no visible spectral differences were identified with respect to the molecular mass of PFO polymers.

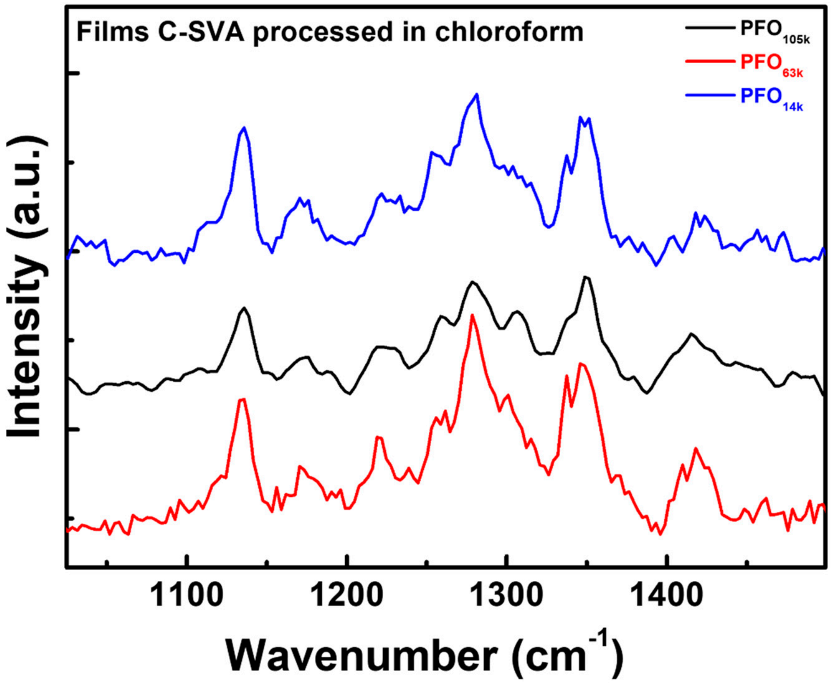

Figure S18. Raman spectra recorded for as spin-cast films of PFO_{14k} (blue), PFO_{63k} (red) and PFO_{105k} (black) systems that were processed via the C-SVA method in chloroform vapors. These spectra are depicting the zoomed-in 1000-1500 cm^{-1} spectral interval. As we can see, no visible spectral differences were identified with respect to the molecular mass of PFO polymers when processed via the C-SVA method.
